# Group sequential analysis of marked point processes: Plasma donation trials

**DOI:** 10.1177/09622802251350263

**Published:** 2025-07-02

**Authors:** Kecheng Li, Richard J Cook

**Affiliations:** 1 Department of Statistics and Actuarial Science, 8430 University of Waterloo, ON, Canada; 2 Michael G. DeGroote Centre for Transfusion Research, 3710 McMaster University, ON, Canada

**Keywords:** Group sequential testing, interim analysis, alpha spending function, heterogeneity, transfusion trial design, correlated binary data, non-inferiority, generalized estimating equation, robust covariance

## Abstract

Plasma donation plays a critical role in modern medicine by providing lifesaving treatments for patients with a wide range of conditions like bleeding disorders, immune deficiencies, and infections. Evaluation of devices used to collect blood plasma from donors is essential to ensure donor safety. We consider the design of plasma donation trials when the goal is to assess the safety of a new device on the response to transfusions compared to the standard device. A unique feature is that the number of donations per donor varies substantially so some individuals contribute more information and others less. The sample size formula is derived to ensure power requirements are met when analyses are based on generalized estimating equations and robust variance estimation. Strategies for interim monitoring based on group sequential designs using alpha spending functions are developed based on a robust covariance matrix for estimates of treatment effect over successive analyses. The design of a plasma donation study is illustrated where the focus is on assessing the safety of a new device with serious hypotensive adverse events as the primary outcome.

## 1. Introduction

Large clinical trials are now routinely designed to incorporate interim analyses to monitor the safety and effectiveness of experimental interventions. Early termination of trials showing unexpected adverse effects of a new treatment minimizes exposure to harm, while early termination when treatments are found to be effective facilitates rapid dissemination of findings and deployment of more useful treatments to affected individuals. In addition to these ethical reasons, trials with planned interim analyses can be expected to reduce costs due to early termination when large treatment effects are seen since fewer participants may need to be recruited and follow-up can be shortened for enrolled participants. The statistical challenge involves control of the experimental type I error rate when conducting repeated significance tests on data accumulating over the course of the study.^1^

In early work on group sequential trials, Pocock^2^ proposed controlling the type I error rate through the derivation of a single critical value, which can be used to guide stopping decisions and determine the statistical significance of the trial results. O’Brien and Fleming^3^ later proposed a design wherein different critical values could be used at successive interim analyses with greater thresholds for early analyses leading to designs which are more conservative for early stopping. These methods all require the specification of a fixed number of interim analyses timed at roughly equal amounts of information under the assumption that data accumulated between successive analysis times are independent of the past data. The so-called independent increment structure enables the use of a recursive integration method^1,4^ to compute standard group sequential boundaries for repeated significance tests. Slud and Wei^5^ generalized the approach to sequential monitoring to allow for departures from the independent increments assumption, greatly expanding the range of analyses for which group sequential analyses could be planned. Lan and DeMets^6^ proposed a more general concept of an alpha spending function which enables a much more flexible and adaptive way to compute critical values for interim tests where the number and timing of analyses can be modified during the course of the study. In trials involving responses based on repeated measures, the independent increments structure for successive test statistics is violated because of the serial dependence of the responses within individuals. Lee and DeMets^7^ proposed to address this for continuous responses based on random-effects models while Wei et al.^8^ and Lee et al.^9^ used generalized estimating equations^10^ to develop a framework applicable to a broader range of data types. Many of these methods however are based on designs in which the repeated measurements are taken at specified fixed time points following randomization.

In many areas interest lies in assessing the effect of an experimental device or intervention which is delivered repeatedly over time but at times determined by the individual and hence which cannot be specified a priori. Examples are ubiquitous and arise in neurology when interest lies in the evaluation of therapies for acute migraine which are only administered when migraines develop,^11^ in respirology where bronchodilators are administered for the resolution of symptoms in recurrent exacerbations in asthma,^12^ and in transfusion medicine where therapeutic transfusion of platelets or red cell products are only given when the need is identified (i.e. platelets are transfused when thrombocytopenia is sufficiently severe that patients are at risk of serious bleeds).^13^ A similar data structure arises in blood donation research in which healthy donors give blood repeatedly over time. Here interest may lie in studying the occurrence of adverse events upon blood donation—in particular in comparing whether two methods of blood collection lead to similar adverse event profiles in donors. In all such settings one can think of the data as arising from a marked point process where the point is the time an intervention is administered and the mark is the response to the intervention. We consider the setting where individuals are randomized to one of two intervention arms and will receive the assigned intervention whenever it is required during the course of follow-up. Our work is motivated by the setting of a blood donation study so here the points are the times that donors arrive at a donation center to give blood and the mark is an indicator of whether an adverse reaction occurred. We consider the setting where the number of donations per donor varies substantially with some individuals contributing more information than others. In particular, we focus on the scenario where donors recruited earlier tend to donate more frequently than those enrolled later. This varying rate of information acquisition introduces a unique dependence structure among successive test statistics.

The remainder of this article is organized as follows. In Section 2, we define notation and give the generalized estimating functions used for estimation and large sample theory for analyses. The joint distribution of the sequential test statistics is then derived. The precise forms of the large sample distributions are given which identify the terms needed to facilitate sample size calculations for a two-arm clinical trial where there is no covariate adjustment and the marginal models for treatment effect simply involve the binary treatment indicator. One element of this large sample distribution involves the distribution of the number of transfusions during the follow-up period. Section 3 proposes the use of a zero-truncated negative binomial process for the need for transfusions. We consider a nuance whereby donors giving more frequent blood donations will tend to be encountered sooner than those donating less frequently. In probabilistic terms, we allow the intensity for transfusions to be higher for those individuals who are recruited earlier in the course of the accrual period—the impact of this is explored in Section 3. Alpha spending functions are reviewed in Section 4. Sample size calculations for the study with interim analyses are given. Both superiority and non-inferiority study designs are considered in Sections 4.2 and 4.3, respectively. Section 5 gives a simulation study demonstrating the good control of the type I error rate and the power for the derived sample sizes. In Section 5.3, we illustrate an example of a plasma donation study design with interim analyses. Discussions and concluding remarks are given in Section 6.

## 2. Statistical formulation and methods

### 2.1. Notation and generalized estimating equations

Let $N_{i}(t)$ denote the cumulative number of donations over a window of calendar time (0,t] for individual i in the donor population of interest. Let $\left\{N_{i}(t),0<t\right\}$ be a right-continuous counting process that records the cumulative number of donations from individual i over the period of calendar time (0,t] with $dN_{i}(t)=N_{i}(t)-N_{i}(t^{-})=1$ indicating a donation took place at calendar time t , and $dN_{i}(t)=0$ otherwise. With the history of donations over (0,t) given by $H_{i}(t)=\{dN_{i}(v),0<v<t\}$ , we assume donations occur in the population according to a mixed Poisson process with conditional intensity^14^

(1) $$\lim_{\Delta t↓0}\frac{P(\Delta N_{i}(t)=1|H_{i}(t),U_{i}=u_{i})}{\Delta t}=u_{i}\lambda (t)$$

where $\Delta N_{i}(t)=N_{i}(t+\Delta t^{-})-N_{i}(t^{-})$ is the number of donations over the interval [t,t+Δt) , $U_{i}>0$ is a random effect with mean one, variance ϕ>0 , and realization $u_{i}$ , and λ(t) is a baseline population average rate function.

**Figure 1.**
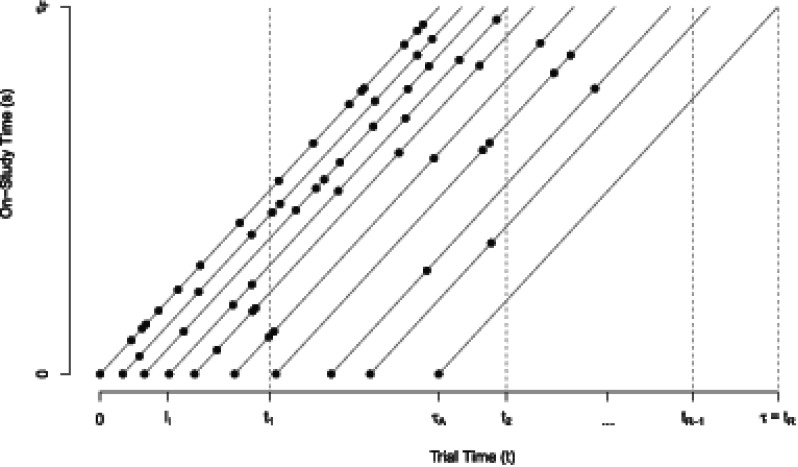
An illustration of the recruitment process for 10 subjects along with their repeated measurements. Interim analyses are planned at study times $t_{1}$ , $t_{2}$ , … , $t_{R-1}$ , and a final analysis at $t_{R}$ . The length of the accrual period is $\tau_{A}$ , the length of the follow-up period is $\tau_{F}$ for each individual, and the entire study duration is $\tau =\tau_{A}+\tau_{F}$ .

We consider a study with an accrual period of length $\tau_{A}$ and a follow-up period for each recruited individual of length $\tau_{F}$ making the total study duration $\tau =\tau_{A}+\tau_{F}$ . It is helpful to consider two time scales with t denoting the time from the start of the accrual period of the trial with 0≤t≤τ and s the time from recruitment for an individual with $0\leq s\leq \tau_{F}$ . We may refer to the former as “trial time” (which is the calendar time with the time origin for t defined as the start of the accrual period), and the latter as “study time” (for time on study for an individual). If $L_{i}$ is the random recruitment time for individual i and $l_{i}$ is its realization then provided $0\leq l_{i}\leq \tau_{A}$ individual i has been on study for a length of time s at $t=l_{i}+s$ . Figure 1 contains a Lexis diagram depicting the accrual and study follow-up periods which define the trial duration, as well as the relation between time-scales t on the horizontal axis and s on the vertical axis. The 45∘ lines emanate from the horizontal axis at the time of recruitment with 10 hypothetical individuals depicted. We assume that the first donation of each subject occurs at their study recruitment time $L_{i}$ . The donation process in (1) plays an important role in study planning as it determines the distribution of the random accrual time $L_{i}$ as well as the associated event intensity during the study period among recruited individuals. In brief the heterogeneity in the rate of donations among individuals in the donor population means that frequent donors will be encountered and recruited early during the accrual period, and as the time from the start of the accrual period passes more donors with lower donation rates will be encountered. This is depicted in Figure 1 by the more frequent donations (represented by the dots on the 45∘ lines) in the individuals recruited earlier compared to individuals recruited later in the period $\left(0,\tau_{A}\right]$ . We consider the implications of this in more detail in Section 3.

Consider the setting where n consenting donors are recruited and randomized upon encountering them during the accrual period. The total number of donations received from the recruited individual i by time t from the start of the trial is

$$K_{i}(t)=\int_{0}^{t}I(l_{i}\leq v\leq l_{i}+\tau_{F})dN_{i}(v)$$

where $I(l_{i}\leq t\leq l_{i}+\tau_{F})$ indicates that individual i is under observation. Let $Y_{ik}$ be the response measured at the k th donation during study follow-up which is observed at time t if $K_{i}(t^{-})=k-1$ and $dK_{i}(t)=1$ ; we can refer to this as the mark associated with the k th donation and here we consider the case where $Y_{ik}$ is a binary response indicating the occurrence of an adverse event where $Y_{ik}=1$ denotes that an adverse event occurs and $Y_{ik}=0$ otherwise. Ultimately we may observe the set of marks $\left\{Y_{ik},k=1,\ldots ,K_{i}(\tau )\right\}$ for individual i , i=1,…,n . Let $X_{i}$ indicate the treatment arm where $X_{i}=1$ if individual i is assigned to the experimental arm and $X_{i}=0$ otherwise. If $V_{ik}$ represents a (p−2)×1 covariate vector measured at the time of the k th donation we let $Z_{ik}=(1,X_{i},V_{ik}^{\prime }{)}^{\prime }$ be the p×1 covariate vector, k=1,2,… , $K_{i}(\tau )$ , i=1,…,n .

Suppose interim analyses are planned at trial times $t_{r}$ , r=1,…,R−1 with a final analysis at study completion (i.e. $t_{R}=\tau$ ); see Figure 1. Let $\Delta_{i}(t_{r})=I(l_{i}\leq t_{r})$ indicate that individual i entered the study by time $t_{r}$ and $n_{r}=\sum_{i=1}^{n}\Delta_{i}(t_{r})$ denote the total number of such individuals contributing to the r th analysis, r=1,…,R , with $n=n_{R}$ . Specifically the vector of responses $Y_{i}(t_{r})=(Y_{i1},\ldots ,Y_{iK_{i}(t_{r})}{)}^{\prime }$ is the set of indicators from the $K_{i}(t_{r})$ donations observed by the time of the r th analysis with $Z_{i}(t_{r})=(Z_{i1}^{\prime },\ldots ,Z_{iK_{i}(t_{r})}^{\prime }{)}^{\prime }$ the corresponding matrix of covariates. The generalized estimating equations^10^ at each interim analysis include subjects recruited by the time of that analysis, so at the r th interim analysis it is given by

(2) $$U(t_{r};\beta ,\alpha )=\sum_{i=1}^{n}U_{i}(t_{r};\beta ,\alpha )=\sum_{i=1}^{n}\Delta_{i}(t_{r})D_{i}^{\prime }(t_{r};\beta )\;\Sigma_{i}(t_{r};\beta ,\alpha {)}^{-1}\;\{Y_{i}(t_{r})-\mu_{i}(t_{r};\beta )\}=0$$

where $\mu_{i}(t_{r};\beta )=(\mu (Z_{i1};\beta ),\ldots ,\mu (Z_{iK_{i}(t_{r})};\beta ){)}^{\prime }$ is the $K_{i}(t_{r})\times 1$ vector of means with

$$\mu (Z_{ik};\beta )=E(Y_{ik}|Z_{ik};\beta )=P(Y_{ik}=1|Z_{ik};\beta ),\;k=1,2,\ldots ,K_{i}(t_{r})$$

indexed by β , $D_{i}(t_{r};\beta )=\partial \mu_{i}(t_{r};\beta )/\partial \beta^{\prime }$ , and $\Sigma_{i}(t_{r};\beta ,\alpha )=\mathrm{cov}(Y_{i}(t_{r})|Z_{i}(t_{r}))$ is the working covariance matrix indexed by a parameter vector α . The marginal probability that $Y_{ik}=1$ is very small in the motivating study since serious hypotensive adverse events are very rare in blood donation.^15^ The natural log link function can therefore be specified under the “Poisson” approximation giving the generalized linear model^16^ of the form

$$\log\;(\mu (Z_{ik};\beta ))=\beta_{0}+\beta_{1}X_{i}+\beta_{2}^{\prime }V_{ik}=\beta^{\prime }Z_{ik}$$

where $\beta =(\beta_{0},\beta_{1},\beta_{2}^{\prime }{)}^{\prime }$ and the estimate of β can be obtained by solving (2). This generalized linear model gives

$$\beta_{1}=\log\;\frac{P(Y_{ik}=1|X_{i}=1,V_{ik};\beta )}{P(Y_{ik}=1|X_{i}=0,V_{ik};\beta )}$$

as the log relative rate of an adverse event for the experimental arm compared to the control arm conditional on the covariate $V_{ik}$ but we note that if $V_{ik}⊥X_{i}$ then this retains the same interpretation if we do not adjust for auxiliary covariates by setting $\beta_{2}=0$ .

The covariance parameter α can be estimated using the moment estimator based on Pearson residuals,^10^ or through second-order estimating equations.^17–19^ Liang and Zeger^10^ showed that $U(t_{r};\beta ,\hat{\alpha })$ is asymptotically equivalent to $U(t_{r};\beta ,\alpha )$ if $\hat{\alpha }$ is $\sqrt{n}$ -consistent, and α is estimated based on the data accumulated by study time $t_{r}$ and updated at each interim analysis. Next we present asymptotic results necessary for developing the joint distribution of sequential test statistics in Section 2.2. By the central limit theorem, we have

(3) $$\frac{1}{\sqrt{n}}\;U(t_{r};\beta ,\alpha )\overset{\;d\;}{\to }\mathrm{MVN}\left(0,B(t_{r})\right),\;\mathrm{as}\;\text{n}\to \infty$$

where

(4) $$B(t_{r})=\mathrm{cov}\left(U_{i}(t_{r};\beta ,\alpha ),U_{i}(t_{r};\beta ,\alpha )\right)=E\left\{U_{i}(t_{r};\beta ,\alpha )U_{i}^{\prime }(t_{r};\beta ,\alpha )\right\}$$

is the p×p asymptotic covariance matrix.

The solution to (2) is the estimate of β at the r th interim analysis and is denoted by $\hat{\beta }(t_{r})$ . Our interest lies primarily in ${\hat{\beta }}_{1}(t_{r})$ , the estimated coefficient of $X_{i}$ , since $\exp\;({\hat{\beta }}_{1}(t_{r}))$ is an estimate of the relative rate of an adverse event in the experimental to control arms. Note

(5) $$\sqrt{n}\;\left(\hat{\beta }(t_{r})-\beta \right)\overset{\;d\;}{\to }\mathrm{MVN}\left(0,\Omega (t_{r})\right),\;\mathrm{as}\;\text{n}\to \infty$$

where the robust covariance matrix is

$$\Omega (t_{r})=A^{-1}(t_{r})\;B(t_{r})\;\{A^{-1}(t_{r}){\}}^{\prime }$$

and

(6) $$A(t_{r})=-E\left\{\frac{\partial }{\partial \beta^{\prime }}U_{i}(t_{r};\beta ,\alpha )\right\}\;=E\left\{\Delta_{i}(t_{r})\;D_{i}^{\prime }(t_{r};\beta )\;\Sigma_{i}(t_{r};\beta ,\alpha {)}^{-1}\;D_{i}(t_{r};\beta )\right\}$$

### 2.2. Joint distribution of sequential test statistics

In this section, we derive the joint distribution of sequential test statistics based on ${\hat{\beta }}_{1}(t_{r})$ , r=1,…,R . Here we are interested in the effect of the experimental device and aim to test

$$H_{0}:\beta_{1}=\beta_{1}\circ \;\;\text{versus}\;\;H_{A}:\beta_{1}\neq \beta_{1}\circ$$

We consider the group sequential setting where analyses are planned at $t_{1}<t_{2}<\cdots <t_{R}=\tau$ . The p -dimensional generalized estimating functions $U(t_{r};\beta ,\alpha )$ in (2) can lead to a pR -dimensional joint multivariate normal distribution of $\left\{n^{-\frac{1}{2}}U(t_{r};\beta ,\alpha ),r=1,\ldots ,R\right\}$ .^9^ Since our primary interest is $\beta_{1}$ and for ease of derivation of the joint distribution of sequential test statistics, we adopt the approach proposed by Lee et al.^9^ in which $U(t_{r};\beta ,\alpha )$ is partitioned as follows:

$${\left(U_{\beta_{1}}(t_{r};\beta ,\alpha ),U_{\theta }^{\prime }(t_{r};\beta ,\alpha )\right)}^{\prime }$$

where $U_{\beta_{1}}(t_{r};\beta ,\alpha )$ is the one-dimensional estimating function for $\beta_{1}$ , and $\theta =(\beta_{0},\beta_{2}^{\prime }{)}^{\prime }$ contains the remaining regression parameters (i.e. excluding $\beta_{1}$ ). Then $U_{\theta }(t_{r};\beta ,\alpha )$ is the (p−1) -dimensional estimating function with respect to θ . Lee et al.^9^ proposed using “profile score function” or profile estimating function of $\beta_{1}$ to construct the sequential test statistics and derive its asymptotic joint distribution. The profile estimating function^9,20^ is written as follows:

$$\bar{U}(t_{r};\beta_{1},\theta ,\alpha )=U_{\beta_{1}}(t_{r};\beta ,\alpha )-A_{\beta_{1}\theta }(t_{r})A_{\theta \theta }^{-1}(t_{r})U_{\theta }(t_{r};\beta ,\alpha )$$

where $A_{\beta_{1}\beta_{1}}(t_{r})$ , $A_{\beta_{1}\theta }(t_{r})$ , $A_{\theta \beta_{1}}(t_{r})$ , and $A_{\theta \theta }(t_{r})$ are submatrices defined conformably with $(\beta_{1},\theta^{\prime }{)}^{\prime }$ as

$$\begin{array}{r} A(t_{r})=\left(\begin{array}{cc} A_{\beta_{1}\beta_{1}}(t_{r}) & A_{\beta_{1}\theta }(t_{r})\\ A_{\theta \beta_{1}}(t_{r}) & A_{\theta \theta }(t_{r}) \end{array}\right) \end{array}$$

By asymptotic multivariate normality and using a linear transformation of (3), we have

(7) $$\frac{1}{\sqrt{n}}\;\bar{U}(t_{r};\beta_{1},\theta ,\alpha )\overset{\;d\;}{\to }N\left(0,M_{1}(t_{r})\right),\;\mathrm{as}\;\text{n}\to \infty$$

with asymptotic variance

$$M_{1}(t_{r})=B_{\beta_{1}\beta_{1}}(t_{r})+A_{\beta_{1}\theta }(t_{r})A_{\theta \theta }^{-1}(t_{r})B_{\theta \theta }(t_{r})[A_{\theta \theta }^{-1}(t_{r}){]}^{{}^{\prime }}[A_{\beta_{1}\theta }(t_{r}){]}^{{}^{\prime }}-2A_{\beta_{1}\theta }(t_{r})A_{\theta \theta }^{-1}(t_{r})B_{\theta \beta_{1}}(t_{r})$$

where $B_{\beta_{1}\beta_{1}}(t_{r})$ , $B_{\beta_{1}\theta }(t_{r})$ , $B_{\theta \beta_{1}}(t_{r})$ , and $B_{\theta \theta }(t_{r})$ are submatrices of $B(t_{r})$ , and

$$\begin{array}{r} B(t_{r})=\left(\begin{array}{cc} B_{\beta_{1}\beta_{1}}(t_{r}) & B_{\beta_{1}\theta }(t_{r})\\ B_{\theta \beta_{1}}(t_{r}) & B_{\theta \theta }(t_{r}) \end{array}\right) \end{array}$$

The asymptotic variance $M_{1}(t_{r})$ can be simplified to $A_{\beta_{1}\beta_{1}}(t_{r})-A_{\beta_{1}\theta }(t_{r})A_{\theta \theta }^{-1}(t_{r})A_{\theta \beta_{1}}(t_{r})$ if the working covariance matrix $\Sigma_{i}(t_{r};\beta ,\alpha )$ is correctly specified since in this case $A(t_{r})=B(t_{r})$ .

The R -dimensional vector of profile estimating functions over all analyses times,

(8) $$\left\{\frac{1}{\sqrt{n}}\;\bar{U}(t_{r};\beta_{1},\theta ,\alpha ),r=1,\ldots ,R\right\}$$

is asymptotically multivariate normal with zero mean and covariance at any two study times $t_{r}$ and $t_{s}$ is given by the following equation:

(9) $$\begin{array}{rl} M_{2}(t_{r},t_{s}) & =\mathrm{cov}({\bar{U}}_{i}(t_{r};\beta_{1},\theta ,\alpha ),{\bar{U}}_{i}(t_{s};\beta_{1},\theta ,\alpha ))\\ & =B_{\beta_{1}\beta_{1}}(t_{r},t_{s})+A_{\beta_{1}\theta }(t_{r})A_{\theta \theta }^{-1}(t_{r})B_{\theta \theta }(t_{r},t_{s})[A_{\theta \theta }^{-1}(t_{s}){]}^{{}^{\prime }}[A_{\beta_{1}\theta }(t_{s}){]}^{{}^{\prime }}\\ & \;-B_{\beta_{1}\theta }(t_{r},t_{s})[A_{\theta \theta }^{-1}(t_{s}){]}^{{}^{\prime }}[A_{\beta_{1}\theta }(t_{s}){]}^{{}^{\prime }}-A_{\beta_{1}\theta }(t_{r})A_{\theta \theta }^{-1}(t_{r})B_{\theta \beta_{1}}(t_{r},t_{s}) \end{array}$$

where submatrices $B_{\beta_{1}\beta_{1}}(t_{r},t_{s})$ , $B_{\beta_{1}\theta }(t_{r},t_{s})$ , $B_{\theta \beta_{1}}(t_{r},t_{s})$ , and $B_{\theta \theta }(t_{r},t_{s})$ are from

(10) $$\begin{array}{rl} B(t_{r},t_{s}) & =E\left\{U_{i}(t_{r};\beta ,\alpha )U_{i}^{\prime }(t_{s};\beta ,\alpha )\right\}=\left(\begin{array}{cc} B_{\beta_{1}\beta_{1}}(t_{r},t_{s}) & B_{\beta_{1}\theta }(t_{r},t_{s})\\ B_{\theta \beta_{1}}(t_{r},t_{s}) & B_{\theta \theta }(t_{r},t_{s}) \end{array}\right) \end{array}$$

By Taylor expansion $\sqrt{n}({\hat{\beta }}_{1}(t_{r})-\beta_{1}\circ )$ can be written as follows:

(11) $$\sqrt{n}\left({\hat{\beta }}_{1}(t_{r})-\beta_{1}\circ \right)={\left\{-\frac{1}{n}\frac{\partial \bar{U}(t_{r};\beta_{1},\theta ,\alpha )}{\partial \beta_{1}}\right\}}^{-1}\frac{1}{\sqrt{n}}\bar{U}(t_{r};\beta_{1},\theta ,\alpha )$$

Under the null hypothesis $\beta_{1}=\beta_{1}\circ$ , (7) and (11) lead to

(12) $$\sqrt{n}\left({\hat{\beta }}_{1}(t_{r})-\beta_{1}\circ \right)\overset{\;d\;}{\to }N\left(0,\frac{M_{1}(t_{r})}{M_{3}^{2}(t_{r})}\right),\;\mathrm{as}\;\text{n}\to \infty$$

where

(13) $$M_{3}(t_{r})=E\left\{-\frac{\partial {\bar{U}}_{i}(t_{r};\beta_{1},\theta ,\alpha )}{\partial \beta_{1}}\right\}=A_{\beta_{1}\beta_{1}}(t_{r})-A_{\beta_{1}\theta }(t_{r})A_{\theta \theta }(t_{r}{)}^{-1}A_{\theta \beta_{1}}(t_{r})$$

It can be shown that $M_{1}(t_{r})/M_{3}^{2}(t_{r})=\Omega_{\beta_{1}\beta_{1}}(t_{r})$ , where $\Omega_{\beta_{1}\beta_{1}}(t_{r})$ is the first diagonal entry of the asymptotic variance $\Omega (t_{r})$ in (5) corresponding to $\beta_{1}$ . Based on (8), (11), and (12) the sequential estimators

(14) $$\left\{\sqrt{n}\left({\hat{\beta }}_{1}(t_{r})-\beta_{1}\circ \right),r=1,\ldots ,R\right\}$$

follow a multivariate normal distribution with mean 0 and covariance matrix $\Gamma =(\gamma_{rs})$ , 1≤r≤s≤R , where

$$\begin{array}{r} \gamma_{rs}=\left\{ \begin{array}{lcl} \Omega_{\beta_{1}\beta_{1}}(t_{r}), & & r=s=1,\ldots ,R\\ M_{2}(t_{r},t_{s})/M_{3}^{2}(t_{s}), & & 1\leq r<s\leq R \end{array}\right. \end{array}$$

The test statistic of interest for the r th analysis can then be written as follows:

$$T(t_{r})=\frac{\sqrt{n_{r}}({\hat{\beta }}_{1}(t_{r})-\beta_{1}\circ )}{\sqrt{\Omega_{\beta_{1}\beta_{1}}(t_{r})}},\;\;r=1,\ldots ,R$$

From (14), it can be shown that

(15) $$\{T(t_{r}),r=1,\ldots ,R\}\overset{\;d\;}{\to }\mathrm{MVN}\left(0,\bar{\Gamma }\right)$$

where $\bar{\Gamma }$ is an R×R covariance matrix with (r,s) element

$${\bar{\gamma }}_{rs}=\frac{\gamma_{rs}}{\sqrt{\gamma_{rr}\gamma_{ss}}},\;1\leq r\leq s\leq R$$

We reject $H_{0}$ at the r th interim analysis if the test statistic $T(t_{r})$ exceeds the corresponding critical value. The serial critical values for the sequential test statistics can be obtained either through standard values directly if the independent increments structure (IIS)^21^ of the statistics holds (see Section 2.3), or through the derivation based on the multivariate joint distribution of sequential test statistics as discussed in Section 4.

If we constrain $\beta_{2}=0$ (i.e. do not adjust for covariates) and assume an exchangeable pairwise correlation ρ for responses within individuals, we obtain

$$\mathrm{cov}(Y_{ij},Y_{ik}|X_{i}=x)=\rho \cdot \mathrm{var}(Y_{ij}|X_{i}=x)$$

Then when taking the expectation of the product of estimating functions in (4) and (10) under the working independence covariance matrix $\Sigma_{i}(t_{r};\beta )$ with the Poisson variance function we have

(16) $$\Omega_{\beta_{1}\beta_{1}}(t_{r})=\frac{1}{\pi (t_{r})}\left\{\frac{2V_{0}}{\kappa_{0}(t_{r})\mu_{0}^{2}}\;\left(1+\frac{\delta_{0}(t_{r})\rho }{\kappa_{0}(t_{r})}\right)+\frac{2V_{1}}{\kappa_{1}(t_{r})\mu_{1}^{2}}\;\left(1+\frac{\delta_{1}(t_{r})\rho }{\kappa_{1}(t_{r})}\right)\right\}$$

in a simple two-arm trial with equal allocation $P(X_{i}=1)=P(X_{i}=0)=0.5$ , where $\pi (t_{r})=P(\Delta_{i}(t_{r})=1)$ , $\kappa_{x}(t_{r})=E\{K_{i}(t_{r})|X_{i}=x,\Delta_{i}(t_{r})=1\}$ is the expected number of donations for a subject in group x for the r th interim analysis, $\mu_{x}=\mu (X_{i}=x;\beta )$ , $V_{x}=\mathrm{var}(Y_{ij}|X_{i}=x)$ , $\nu_{x}(t_{r})=\mathrm{var}(K_{i}(t_{r})|X_{i}=x,\Delta_{i}(t_{r})=1)$ is the variance of the number of donations for a subject in group x for the r th interim analysis, and $\delta_{x}(t_{r})=\nu_{x}(t_{r})+\kappa_{x}^{2}(t_{r})-\kappa_{x}(t_{r})$ , x=0,1 . The asymptotic variance of ${\hat{\beta }}_{1}(t_{r})$ is obtained by dividing (16) by $n_{r}$ . See Sections A and B of the Supplemental Material for the details of the derivation of (16).

### 2.3. The independent increments structure

The independent increments structure (IIS) refers to a property of sequential test statistics where the random variables providing data between the current analysis time at $t_{r}$ and the next analysis time $t_{r+1}$ are statistically independent of random variables providing the current data.^21^ Lee et al.^9^ proved that $n^{-\frac{1}{2}}\bar{U}(t_{r};\beta_{1},\theta ,\alpha )$ has the asymptotic IIS if the covariance function $\Sigma (t_{r};\beta ,\alpha )$ is correctly specified. Based on (11), the asymptotically IIS of the sequentially computed test statistics can be assessed by the following equation:

(17) $$M_{1}(t_{r})=M_{2}(t_{r},t_{s}),\;r<s$$

through

$$\mathrm{cov}(n^{-\frac{1}{2}}\bar{U}(t_{r};\beta_{1},\theta ,\alpha ),n^{-\frac{1}{2}}\bar{U}(t_{s};\beta_{1},\theta ,\alpha ))=\mathrm{var}(n^{-\frac{1}{2}}\bar{U}(t_{r};\beta_{1},\theta ,\alpha )),\;r<s$$

If the independent increments property holds then the established critical values for standard group sequential boundaries of sequential test statistics can be used; these are computed through recursive univariate integration as described by Armitage et al.^1^ If the IIS does not hold, the joint distribution of the sequential test statistics, and in particular the covariance matrix of successive test statistics in (15) must be used to obtain critical values which enables control of the type I error rate. We show how this can be carried out in practice in Section 4.

## 3. Modeling the accrual time and subsequent donation process

As shown in (16) in Section 2.2, the mean and variance of the number of measurements per individual are required to evaluate the joint distribution of the sequential test statistics for the sample size calculation. Suppose the number of donations per individual over a period of duration τ is N(τ) . As noted in Section 2.1, if the number of donations per individual varies according to a mixed time-homogeneous Poisson process, then conditionally on U , N(τ)∣U=u∼Poisson(uλτ) , and

$$P(N(\tau )=k|u)=(u\lambda \tau {)}^{k}e^{-u\lambda \tau }/k!$$

where E(U)=1 and var(U)=ϕ . We consider a negative binomial process for which $U∼\mathrm{Gamma}(\phi^{-1},\phi )$ . Suppose the recruitment time of a random subject in the population given its random effect follows an exponential distribution with

L∣U=u∼Exp(uλ)

where the random effect $U∼\mathrm{Gamma}(\phi^{-1},\phi )$ . We note that subjects who have a higher frequency of donations in calendar time will tend to be recruited earlier in the accrual period. Thus for the trial design with interim monitoring we need to consider the recruitment time of the individual when modeling the number of donations they have by each interim analysis time. The conditional distribution of the random effect U given recruitment time L=l then follows a modified gamma distribution with

$$U|L=l∼\mathrm{Gamma}\left(\phi^{-1}+1,\frac{\phi }{\phi \;\lambda \;l+1}\right)$$

With E(U∣L=l)=(1+ϕ)/(1+ϕλl) this reflects the fact that individuals with an earlier recruitment time tend to have larger random effects, which contributes to them providing a larger number of repeated measurements during the follow-up period compared to those recruited at a later time.

To compute the expected number of donations over the course of the trial to time $t_{r}$ we average over the recruitment time for recruited individuals. The mean of the number of donations per individual by study time $t_{r}$ with $X_{i}=x$ is denoted by $\kappa_{x}(t_{r})$ and given as follows:

$$\begin{array}{rl} \kappa_{x}(t_{r}) & =E\{K_{i}(t_{r})|X_{i}=x,\Delta_{i}(t_{r})=1\}\\ & =E_{L}\{E_{U}\{E\{K_{i}(t_{r})|X_{i}=x,\Delta_{i}(t_{r})=1,\\ & \;U_{i}=u,L_{i}=l\}|\Delta_{i}(t_{r})=1,L_{i}<\min(t_{r},\tau_{A})\}|\Delta_{i}(t_{r})=1,L_{i}<\min(t_{r},\tau_{A})\} \end{array}$$

The variance of the number of donations per individual by study time $t_{r}$ , $\nu_{x}(t_{r})$ , can then be obtained as $E\{K_{i}^{2}(t_{r})|X_{i}=x,\Delta_{i}(t_{r})=1\}-\kappa_{x}^{2}(t_{r})$ , which is a function of λ , ϕ , $\tau_{A}$ , $\tau_{F}$ , and $t_{r}$ . Similarly, the mean number of donations per individual between study time $t_{r}$ and $t_{s}$ in group x can be calculated via $\kappa_{x}(t_{r},t_{s})=E\{K_{i}(t_{r},t_{s})|X_{i}=x,\Delta_{i}(t_{r})=1\}$ , where $K_{i}(t_{r},t_{s})=K_{i}(t_{s})-K_{i}(t_{r})$ , $0<t_{r}<t_{s}\leq \tau$ . The explicit forms of $\kappa_{x}(t_{r})$ , $\nu_{x}(t_{r})$ , and $\kappa_{x}(t_{r},t_{s})$ with detailed derivations are given in (C.10) to (C.16) in Supplemental Material C. Since the number of donations per recruited individual does not depend on the treatment arms here we have $\kappa (t_{r})=\kappa_{x}(t_{r})$ , $\nu (t_{r})=\nu_{x}(t_{r})$ , and $\kappa (t_{r},t_{s})=\kappa_{x}(t_{r},t_{s})$ , x=0,1 . With $\kappa (t_{r})$ , $\nu (t_{r})$ , and $\kappa (t_{r},t_{s})$ derived we can evaluate the joint distribution of sequential test statistics which is the key for the calculations of the group sequential boundaries and sample size for interim monitoring.

For a study design with a single analysis at study completion (i.e. a study without interim analysis), let $K_{A}$ denote the number of donations from a random subject during the accrual period of length $\tau_{A}$ , and $K_{F}$ be the number of donations from the subject in the follow-up window of length $\tau_{F}$ . Li^15^ has shown that the mean of $K_{F}$ given that subject has at least one donation during the recruitment phase ( $K_{A}\geq 1$ ) is

$$E(K_{F}|K_{A}\geq 1)=\lambda \tau_{F}\left\{\frac{1-(1+\lambda \tau_{A}\phi {)}^{-\phi^{-1}-1}}{1-(1+\lambda \tau_{A}\phi {)}^{-\phi^{-1}}\;}\right\}$$

and the variance of $K_{F}$ given $K_{A}\geq 1$ is

$$\mathrm{var}(K_{F}|K_{A}\geq 1)=E(K_{F}|K_{A}\geq 1)-E^{2}(K_{F}|K_{A}\geq 1)+(\lambda \tau_{F}{)}^{2}(1+\phi )\left\{\frac{1-(1+\lambda \tau_{A}\phi {)}^{-\phi^{-1}-2}}{1-(1+\lambda \tau_{A}\phi {)}^{-\phi^{-1}}}\right\}$$

It can be shown that $\kappa (\tau )=E(K_{F}|K_{A}\geq 1)$ and $\nu (\tau )=\mathrm{var}(K_{F}|K_{A}\geq 1)$ which means that in a sequential study that does not terminate early, the expected number of donations per individual by the end of the study and their variance remain the same as in a fixed study without interim analysis. Hence the modeling of the recruitment time on number of donations is a special case for the design of sequential analysis.

## 4. Sample size and group sequential testing

### 4.1. Alpha spending functions

Let $C(t_{r})$ be the critical value or group sequential boundary at $t_{r}$ such that if $|T(t_{r})|>C(t_{r})$ we reject $H_{0}$ and may consider the termination of the study. Let $\alpha^{*}(t)$ be an increasing function with $\alpha^{*}(0)=0$ and $\alpha^{*}(1)=\alpha$ where α is the predetermined significance level. The alpha spending function $\alpha^{*}(t)$ proposed by Lan and DeMets^6^ allocated the significance level α over the interim analyses, and based on the alpha spending function the group sequential boundaries can be determined by solving

$$\begin{array}{rl} P(|T(t_{1})| & >C(t_{1});\;\beta_{1}=\beta_{1}\circ )+P(|T(t_{1}))|\leq C(t_{1}),\;|T(t_{2})|>C(t_{2});\;\beta_{1}=\beta_{1}\circ )+\ldots \\ & \;+P(|T(t_{r})|\leq C(t_{r}),\;r=1,\ldots ,R-1,\;|T(t_{R})|>C(t_{R});\;\beta_{1}=\beta_{1}\circ )=\alpha^{*}(\omega_{R}) \end{array}$$

or

(18) $$\begin{array}{rl} & P(|T(t_{1})|>C(t_{1});\;\beta_{1}=\beta_{1}\circ )=\alpha^{*}(\omega_{1})\;,\text{ and} \end{array}$$

$$\begin{array}{rl} & P(|T(t_{v})|\leq C(t_{v}),v=1,\ldots ,r-1,\;|T(t_{r})|>C(t_{r});\;\beta_{1}=\beta_{1}\circ )=\alpha^{*}(\omega_{r})-\alpha^{*}(\omega_{r-1})\;,\;r=2,\ldots ,R \end{array}$$

where $\omega_{r}$ is the information fraction for the r th interim analysis, $0\leq \omega_{r}\leq 1$ . There are three common ways to define the information fraction. The first is using the statistical information which is the inverse of the variance of the estimated effect (e.g. $n_{r}/\Omega_{\beta_{1}\beta_{1}}(t_{r})$ for the r th interim analysis). If the maximum information is known or can be specified at the design stage based on historical research, the information fraction can be defined as $\omega_{r}=I(t_{r})/I(\tau )$ , where $I(t_{r})=n_{r}/\Omega_{\beta_{1}\beta_{1}}(t_{r})$ . This can also be used as the guidance of determining the time of interim analysis. If the maximum information is unknown we can instead use the second approach based on the expected number of subjects/measurements (i.e. $\omega_{r}=n_{r}/n$ ), The third is to specify it in terms of the expected study duration (i.e. $\omega_{r}=t_{r}/\tau )$ .

Lan and DeMets^6^ proposed the use of alpha spending functions which allow for flexible interim monitoring of clinical trials. Examples of commonly used alpha spending functions include

$$\begin{array}{rl} \alpha_{1}^{*}(t) & =2-2\Phi (Z_{\alpha /2}/\sqrt{t})\\ \alpha_{2}^{*}(t) & =\alpha \log\;\{1+(e-1)t\}\\ \alpha_{3}^{*}(t) & =\alpha t \end{array}$$

The boundaries generated by function $\alpha_{1}^{*}(t)$ and $\alpha_{2}^{*}(t)$ are similar to the boundaries obtained by O’Brien and Fleming^3^ and Pocock,^2^ respectively, and we refer them to the O’Brien-Fleming and Pocock type alpha spending functions, respectively. The third one $\alpha_{3}^{*}(t)$ is the uniform alpha spending function as it allocates the type I error rate evenly over time.^6^

### 4.2. Sample size calculation for a sequential superiority trial

For a superiority trial, we consider a two-sided test with the aim to test

$$H_{0}:\beta_{1}=\beta_{1}^{\circ }=0\text{ versus }H_{A}:\beta_{1}\neq 0$$

where the true effect is denoted as $\beta_{1A}$ . Interest lies in one direction (i.e. typically $\beta_{1A}<0$ ) but we consider a two-sided test as is standard practice, and conventionally we take the probability of rejection in the opposite direction to the true effect as negligible. Using the joint distribution of the sequential test statistics in (15) and assumptions about the response process, accrual, and transfusions discussed in Sections 2.1 and 3, it is possible to determine the times of interim analyses that will give a dependence structure aligned with the independent increments property by verifying (17). This will enable the use of established critical values directly.^9^ The times of interim analysis can also be determined based on the proportions of the maximum information, for example, times corresponding to 1/3, 2/3 of the maximum information for a study with two interim analyses and one final analysis. In such cases or more generally for analyses planned at any key times of interest, the critical values can be derived through (18) to ensure the control of the type I error rate at α for the specified alpha spending function. The sample size for a sequential superiority study can be calculated by solving (19) with the desired power,

(19) $$\begin{array}{rl} \mathrm{Power} & =P\left(\;|T(t_{1})|>C(t_{1});\beta_{1}=\beta_{1A}\right)+P\left(\;|T(t_{1})|\leq C(t_{1}),|T(t_{2})|>C(t_{2});\beta_{1}=\beta_{1A}\right)\\ & \;+\cdots +P\left(\;|T(t_{r})|\leq C(t_{r}),r=1,\ldots ,R-1,|T(t_{R})|>C(t_{R});\beta_{1}=\beta_{1A}\right) \end{array}$$

### 4.3. Sample size calculation for a sequential non-inferiority trial

For a non-inferiority design, we consider a one-sided test with the aim to test

$$H_{0}:\beta_{1}\geq \beta_{1M}\;\;\text{versus}\;\;H_{A}:\beta_{1}<\beta_{1M}$$

where $\beta_{1M}$ is the non-inferiority margin of $\beta_{1}$ , that is, $\beta_{1M}=\log\;RR_{M}$ , and for $RR<RR_{M}$ we can claim non-inferiority on the relative rate scale. This corresponds to the case where the relative rate of an adverse event for the blood donation using the experimental system versus the standard system is no greater than $RR_{M}$ . Based on (18), the critical values for the sequential tests for the non-inferiority trial can be solved through

$$\begin{array}{rl} & P(T(t_{1})<-C(t_{1});\;\beta_{1}=\beta_{1}\circ )=\alpha^{*}(\omega_{1})\;,\;r=1\;,\text{ and }P(-C(t_{v})\leq T(t_{v})\leq 0,v=1,\ldots ,r-1,\;T(t_{r})<-C(t_{r});\;\beta_{1}=\beta_{1}\circ )\\ & \;=\alpha^{*}(\omega_{r})-\alpha^{*}(\omega_{r-1})\;,\;r=2,\ldots ,R \end{array}$$

Once the critical values have been determined, the sample size for the study with interim analyses can be calculated by solving (20) such that the desired power requirement is met,

(20) $$\begin{array}{rl} \mathrm{Power}= & P\left(\;T(t_{1})<-C(t_{1});\beta_{1}=\beta_{1A}\right)\\ & +P\left(\;-C(t_{1})\leq T(t_{1})\leq 0,\;T(t_{2})<-C(t_{2});\beta_{1}=\beta_{1A}\right)\\ & +\cdots +P\left(\;-C(t_{r})\leq T(t_{r})\leq 0,r=1,\ldots ,R-1,\;T(t_{R})<-C(t_{R});\beta_{1}=\beta_{1A}\right) \end{array}$$

## 5. Simulation studies

### 5.1. Empirical validation with sequential trials

In this section, we report on the results of simulation studies designed to validate the derivations. Let λ=10,18 , ϕ=0.8,1.6 , $\tau_{A}=0.5$ , $\tau_{F}=1$ , ρ=0.005,0.01 , $\mu_{0}=0.002$ , $\beta_{1A}=\log\;(1.2)$ , and type I error rates are controlled at the significance level of α=5% . Also let $\beta_{1}\circ =0$ for the superiority study, and $\beta_{1M}=\log\;(1.5)$ for the non-inferiority study. The sample sizes are then calculated to ensure adequate power of trials (at least 80% or 90%). Suppose we plan for two interim analyses with a final analysis at the end of the study so R=3 . The times of two interim analyses are determined based on the proportions of the maximum information where the first and second analysis are at the times corresponding to 1/3 and 2/3 of the maximum information, and the times of interim analyses can be determined without knowing the actual maximum information but the asymptotic variance of $\beta_{1}$ , that is, $\Omega_{\beta_{1}\beta_{1}}(t_{r})/n_{r}$ , and the probability of being recruited by time $t_{r}$ , that is, $\pi (t_{r})$ . In this example, suppose the information for the two interim analyses is the inverse of the asymptotic variance of $\beta_{1}$ , that is,

$$I(t_{1})=\frac{n_{1}}{\Omega_{\beta_{1}\beta_{1}}(t_{1})}=\frac{\pi (t_{1})n}{\Omega_{\beta_{1}\beta_{1}}(t_{1})}\;\text{and}\;I(t_{2})=\frac{n_{2}}{\Omega_{\beta_{1}\beta_{1}}(t_{2})}=\frac{\pi (t_{2})n}{\Omega_{\beta_{1}\beta_{1}}(t_{2})}$$

and the maximum information at the end of the study is $I(\tau )=n/\Omega_{\beta_{1}\beta_{1}}(\tau )$ . By taking the ratio of the information at $t_{1}$ and $t_{2}$ with the maximum information, respectively, gives two information fractions (i.e. $I(t_{1})/I(\tau )$ and $I(t_{2})/I(\tau )$ ), and the times of two interim analyses are chosen at the times such that the information fractions equal to 1/3 and 2/3, respectively, that is, $t_{1}$ and $t_{2}$ are determined such that

$$\frac{I(t_{1})}{I(\tau )}=\frac{\pi (t_{1})\Omega_{\beta_{1}\beta_{1}}(\tau )}{\Omega_{\beta_{1}\beta_{1}}(t_{1})}=\frac{1}{3}\;\text{and}\;\frac{I(t_{2})}{I(\tau )}=\frac{\pi (t_{2})\Omega_{\beta_{1}\beta_{1}}(\tau )}{\Omega_{\beta_{1}\beta_{1}}(t_{2})}=\frac{2}{3}$$

where

$$\pi (t_{r})=\frac{1-(\lambda \phi t_{r}+1{)}^{-\phi^{-1}}}{1-(\lambda \phi \tau_{A}+1{)}^{-\phi^{-1}}}$$

Once the interim analyses times are determined, the group sequential boundaries can be derived and sample sizes needed for the study with two interim analyses can be calculated as described in Sections 4.2 and 4.3. Three different alpha spending functions are considered. The required sample sizes for each scenario are provided in Table 1 for the superiority study design, and Table 2 for the non-inferiority study. The “Fixed study” columns of Tables 1 and 2 record the sample sizes required for a fixed study without any interim analysis. The columns after “Fixed study” record the sample sizes required for the study with interim monitoring using different alpha spending functions. The sample size needed for a study with interim analyses increases compared to a fixed sample size study in order to reach the same desired power since the critical value is higher.

**Table 1.** Sample size required in terms of the number of subjects needed for a superiority design with $\mu_{0}=0.002$ , $\exp\;(\beta_{1A})=1.2$ , $\tau_{A}=0.5$ , $\tau_{F}=1$ , and $\beta_{1}\circ =0$ ; two interim analyses are planned with R=3 . The average sample size required for the sequential trial under each scenario and spending function based on N=1000 simulations is reported in parentheses.

					80% Power				90% Power			
						Interim monitoring with				Interim monitoring with		
						alpha spending function				alpha spending function		
λ	ϕ	ρ	κ	ν	Fixed study	$\alpha_{1}^{*}(t)$	$\alpha_{2}^{*}(t)$	$\alpha_{3}^{*}(t)$	Fixed study	$\alpha_{1}^{*}(t)$	$\alpha_{2}^{*}(t)$	$\alpha_{3}^{*}(t)$
10	0.80	0.005	12.24	91.69	38,610	39,530	45,241	43,199	51,687	52,823	59,734	57,276
						(39,405)	(44,621)	(42,704)		(52,579)	(58,711)	(56,355)
10	0.80	0.010	12.24	91.69	41,915	42,811	49,014	46,790	56,113	57,219	64,710	62,033
						(42,719)	(48,035)	(45,907)		(56,962)	(63,007)	(60,626)
10	1.60	0.005	14.01	190.72	34,923	35,755	40,919	39,073	46,752	47,782	54,027	51,803
						(35,629)	(40,244)	(38,514)		(47,541)	(52,834)	(50,864)
10	1.60	0.010	14.01	190.72	39,026	39,863	45,633	43,567	52,244	53,280	60,244	57,758
						(39,741)	(44,426)	(42,843)		(52,927)	(58,100)	(56,108)
18	0.80	0.005	20.23	277.35	24,872	25,421	29,107	27,788	33,296	33,981	38,427	36,840
						(25,378)	(28,752)	(27,521)		(33,883)	(37,806)	(36,342)
18	0.80	0.010	20.23	277.35	28,389	28,965	33,155	31,651	38,004	38,716	43,770	41,961
						(28,931)	(32,613)	(31,267)		(38,570)	(42,786)	(41,228)
18	1.60	0.005	22.72	577.77	23,493	24,009	27,482	26,237	31,450	32,087	36,283	34,783
						(23,965)	(26,967)	(25,851)		(31,938)	(35,424)	(34,072)
18	1.60	0.010	22.72	577.77	27,975	28,526	32,636	31,157	37,450	38,129	43,080	41,306
						(28,460)	(31,806)	(30,476)		(37,958)	(41,619)	(40,182)

**Table 2.** Sample size required in terms of the number of subjects needed for a non-inferiority design with $\mu_{0}=0.002$ , $\exp\;(\beta_{1A})=1.2$ , $\tau_{A}=0.5$ , $\tau_{F}=1$ , and non-inferiority margin $\exp\;(\beta_{1M})=1.5$ ; two interim analyses are planned with R=3 . The average sample size required for the sequential trial under each scenario and spending function based on N=1000 simulations is reported in parentheses.

					80% Power				90% Power			
						Interim monitoring with				Interim monitoring with		
						alpha spending function				alpha spending function		
λ	ϕ	ρ	κ	ν	Fixed study	$\alpha_{1}^{*}(t)$	$\alpha_{2}^{*}(t)$	$\alpha_{3}^{*}(t)$	Fixed study	$\alpha_{1}^{*}(t)$	$\alpha_{2}^{*}(t)$	$\alpha_{3}^{*}(t)$
10	0.80	0.005	12.24	91.69	20,303	20,748	23,913	22,758	28,123	28,692	32,640	31,206
						(20,699)	(23,546)	(22,471)		(28,578)	(32,036)	(30,695)
10	0.80	0.010	12.24	91.69	22,042	22,473	25,918	24,658	30,531	31,079	35,373	33,808
						(22,422)	(25,385)	(24,189)		(30,929)	(34,408)	(32,945)
10	1.60	0.005	14.01	190.72	18,365	18,766	21,628	20,584	25,438	25,951	29,522	28,225
						(18,706)	(21,271)	(20,272)		(25,836)	(28,853)	(27,695)
10	1.60	0.010	14.01	190.72	20,522	20,927	24,128	22,956	28,426	28,944	32,926	31,475
						(20,862)	(23,515)	(22,455)		(28,749)	(31,798)	(30,514)
18	0.80	0.005	20.23	277.35	13,079	13,345	15,390	14,642	18,117	18,456	21,004	20,077
						(13,326)	(15,183)	(14,481)		(18,406)	(20,649)	(19,761)
18	0.80	0.010	20.23	277.35	14,929	15,205	17,535	16,681	20,678	21,029	23,928	22,869
						(15,172)	(17,211)	(16,453)		(20,978)	(23,344)	(22,409)
18	1.60	0.005	22.72	577.77	12,354	12,602	14,530	13,825	17,112	17,429	19,831	18,955
						(12,567)	(14,252)	(13,615)		(17,366)	(19,343)	(18,557)
18	1.60	0.010	22.72	577.77	14,711	14,976	17,257	16,418	20,377	20,710	23,549	22,509
						(14,945)	(16,826)	(16,066)		(20,607)	(22,730)	(21,797)

**Table 3.** Empirical rejection rates for a superiority trial with two interim analyses.

				$\alpha_{1}^{*}(t)$				$\alpha_{2}^{*}(t)$				$\alpha_{3}^{*}(t)$			
				80% Power		90% Power		80% Power		90% Power		80% Power		90% Power	
				Emp. type	Emp.	Emp. type	Emp.	Emp. type	Emp.	Emp. type	Emp.	Emp. type	Emp.	Emp. type	Emp.
λ	ϕ	ρ	t	I error	power	I error	power	I error	power	I error	power	I error	power	I error	power
10	0.8	0.005	$t_{1}$	0.0	8.4	0.2	12.3	3.0	36.5	1.9	45.8	1.5	30.7	1.6	43.0
			$t_{2}$	1.9	42.9	1.9	56.0	2.0	24.8	1.2	31.6	1.5	28.3	1.1	32.9
			$t_{3}$	3.3	29.6	3.3	22.7	0.7	16.3	0.7	11.8	1.2	21.1	1.8	13.7
			Total	5.2	80.9	5.4	91.0	5.7	77.6	3.8	89.2	4.2	80.1	4.5	89.6
10	0.8	0.01	$t_{1}$	0.1	3.4	0.0	7.1	2.6	31.8	1.9	41.8	0.9	30.1	1.9	36.1
			$t_{2}$	2.1	44.5	1.2	56.0	1.5	29.1	1.7	34.2	1.6	29.4	2.8	36.2
			$t_{3}$	4.0	32.5	3.4	26.8	1.2	16.5	1.3	14.7	1.5	21.1	0.9	17.6
			Total	6.2	80.4	4.6	89.9	5.3	77.4	4.9	90.7	4.0	80.6	5.6	89.9
10	1.6	0.005	$t_{1}$	0.1	8.1	0.3	11.3	2.0	37.6	2.7	50.3	1.6	32.7	1.5	41.3
			$t_{2}$	2.2	42.1	1.7	54.2	2.1	27.3	1.5	26.6	1.7	28.8	1.0	35.5
			$t_{3}$	1.8	28.0	2.9	23.6	1.5	14.9	1.1	13.6	2.0	17.5	2.0	14.5
			Total	4.1	78.2	4.9	89.1	5.6	79.8	5.3	90.5	5.3	79.0	4.5	91.3
10	1.6	0.01	$t_{1}$	0.1	4.3	0.2	9.1	2.2	36.6	2.4	49.3	1.7	23.0	1.4	39.5
			$t_{2}$	1.3	47.4	1.8	51.8	2.5	28.4	1.6	27.5	1.8	32.8	2.0	34.7
			$t_{3}$	3.9	29.3	2.6	28.9	0.9	16.8	1.8	13.3	1.9	20.7	1.2	14.5
			Total	5.3	81.0	4.6	89.8	5.6	81.8	5.8	90.1	5.4	76.5	4.6	88.7
18	0.8	0.005	$t_{1}$	0.1	5.1	0.0	7.8	1.6	34.0	2.5	45.0	1.8	26.7	1.4	37.8
			$t_{2}$	0.9	45.0	1.0	56.2	1.7	30.0	1.8	31.4	1.8	34.2	1.7	35.6
			$t_{3}$	3.0	30.6	2.6	25.6	1.2	17.6	0.5	13.0	1.7	18.8	0.9	16.5
			Total	4.0	80.7	3.6	89.6	4.5	81.6	4.8	89.4	5.3	79.7	4.0	89.9
18	0.8	0.01	$t_{1}$	0.0	2.1	0.0	6.4	2.2	29.6	2.7	40.5	1.6	21.8	1.3	31.6
			$t_{2}$	1.2	42.9	1.9	58.0	1.9	29.7	2.1	33.4	1.2	33.0	1.6	40.8
			$t_{3}$	3.7	34.4	2.9	28.0	0.9	20.0	1.6	15.5	1.3	23.3	1.1	17.3
			Total	4.9	79.4	4.8	92.4	5.0	79.3	6.4	89.4	4.1	78.1	4.0	89.7
18	1.6	0.005	$t_{1}$	0.0	3.8	0.0	9.1	1.8	37.3	2.5	47.0	1.6	29.4	1.1	40.8
			$t_{2}$	1.8	47.1	1.5	57.5	1.3	25.9	1.9	29.5	1.7	32.4	1.7	37.3
			$t_{3}$	2.6	29.0	3.2	23.6	0.8	17.0	0.9	12.3	1.8	19.4	1.4	13.1
			Total	4.4	79.9	4.7	90.2	3.9	80.2	5.3	88.8	5.1	81.2	4.2	91.2
18	1.6	0.01	$t_{1}$	0.0	3.1	0.1	5.7	1.7	33.9	3.4	45.3	1.2	29.3	1.4	36.3
			$t_{2}$	2.4	48.6	1.3	58.5	1.1	30.3	1.7	31.7	1.6	33.9	1.7	36.6
			$t_{3}$	3.9	30.5	3.5	27.3	0.9	16.2	0.7	13.7	1.3	17.2	0.9	16.4
			Total	6.3	82.2	4.9	91.5	3.7	80.4	5.8	90.7	4.1	80.4	4.0	89.3

**Table 4.** Empirical rejection rates for a non-inferiority trial with two interim analyses.

				$\alpha_{1}^{*}(t)$				$\alpha_{2}^{*}(t)$				$\alpha_{3}^{*}(t)$			
				80% Power		90% Power		80% Power		90% Power		80% Power		90% Power	
				Emp. type	Emp.	Emp. type	Emp.	Emp. type	Emp.	Emp. type	Emp.	Emp. type	Emp.	Emp. type	Emp.
λ	ϕ	ρ	t	I error	power	I error	power	I error	power	I error	power	I error	power	I error	power
10	0.8	0.005	$t_{1}$	0.0	6.4	0.0	10.7	2.8	40.9	2.0	49.6	1.1	33.7	1.5	43.8
			$t_{2}$	2.6	45.4	1.7	52.0	1.5	26.9	2.1	27.4	1.6	30.4	1.2	31.4
			$t_{3}$	3.1	27.4	2.4	25.5	0.9	14.0	1.1	12.4	1.6	16.8	1.8	14.9
			Total	5.7	79.2	4.1	88.2	5.2	81.8	5.2	89.4	4.3	80.9	4.5	90.1
10	0.8	0.01	$t_{1}$	0.2	3.6	0.0	8.0	1.2	32.7	1.6	43.4	1.8	30.3	1.4	40.6
			$t_{2}$	2.3	41.4	1.5	52.8	1.2	29.5	1.5	29.6	1.7	32.2	2.1	32.3
			$t_{3}$	2.7	33.9	4.0	28.6	1.0	15.9	1.6	14.7	1.8	17.0	1.5	14.9
			Total	5.2	78.9	5.5	89.4	3.4	78.1	4.7	87.7	5.3	79.5	5.0	87.8
10	1.6	0.005	$t_{1}$	0.0	7.5	0.1	10.5	3.2	37.6	1.0	51.7	1.0	34.6	1.7	42.9
			$t_{2}$	2.5	43.8	2.1	55.5	0.7	25.9	0.9	27.8	1.7	27.9	1.5	33.4
			$t_{3}$	3.9	27.6	2.3	23.1	0.6	15.9	0.9	9.9	2.3	18.3	1.3	14.3
			Total	6.4	78.9	4.5	89.1	4.5	79.4	2.8	89.4	5.0	80.8	4.5	90.6
10	1.6	0.01	$t_{1}$	0.1	4.3	0.0	9.2	3.0	35.2	2.0	47.6	2.0	30.2	1.2	42.3
			$t_{2}$	1.7	45.0	2.4	56.2	1.6	30.5	2.1	29.0	1.3	30.3	1.7	35.5
			$t_{3}$	2.2	31.9	3.4	23.7	1.2	15.3	0.9	10.6	0.7	18.6	1.7	12.7
			Total	4.0	81.2	5.8	89.1	5.8	81.0	5.0	87.2	4.0	79.1	4.6	90.5
18	0.8	0.005	$t_{1}$	0.2	4.2	0.1	7.7	1.5	37.2	2.4	47.1	1.5	30.6	1.7	43.7
			$t_{2}$	1.5	45.5	1.4	55.4	1.4	25.5	1.8	31.0	1.9	29.0	1.0	32.2
			$t_{3}$	3.5	32.6	2.4	28.2	1.4	16.2	1.1	13.0	1.3	19.3	1.5	14.7
			Total	5.2	82.3	3.9	91.3	4.3	78.9	5.3	91.1	4.7	78.9	4.2	90.6
18	0.8	0.01	$t_{1}$	0.1	3.9	0.0	4.5	2.6	33.4	2.7	44.2	2.4	24.7	1.5	36.2
			$t_{2}$	1.0	42.6	1.8	53.7	1.7	27.1	1.5	31.7	1.8	32.4	1.5	38.6
			$t_{3}$	4.2	33.0	4.1	30.0	1.2	18.4	1.0	13.9	1.6	20.4	0.8	15.5
			Total	5.3	79.5	5.9	88.2	5.5	78.9	5.2	89.8	5.8	77.6	3.8	90.3
18	1.6	0.005	$t_{1}$	0.0	5.7	0.1	7.3	2.1	38.1	3.9	49.0	2.0	30.3	1.2	41.7
			$t_{2}$	1.6	43.4	2.2	56.4	1.5	26.7	1.7	28.8	2.0	30.4	2.1	35.2
			$t_{3}$	2.9	30.8	3.0	24.8	1.6	14.1	0.8	11.1	1.3	18.9	1.0	13.1
			Total	4.5	79.9	5.3	88.5	5.2	78.9	6.4	88.9	5.3	79.6	4.3	90.0
18	1.6	0.01	$t_{1}$	0.0	2.9	0.1	6.7	1.6	33.4	2.2	46.4	1.7	28.8	1.7	42.2
			$t_{2}$	1.1	48.2	1.5	56.6	2.6	30.5	1.7	33.0	1.5	31.3	1.4	31.3
			$t_{3}$	4.4	30.7	4.1	27.1	1.2	15.7	0.8	11.6	0.7	17.7	1.8	15.1
			Total	5.5	81.8	5.7	90.4	5.4	79.6	4.7	91.0	3.9	77.8	4.9	88.6

With the calculated sample size for study with the planned interim analyses for each scenario we assess the empirical rejection rates to verify the sample size calculation. Under each parameter setting, for each subject i we generate random effect $U_{i}∼\mathrm{Gamma}(\phi^{-1},\phi )$ and treatment assignment $X_{i}∼\mathrm{Bernoulli}(0.5)$ . The recruitment time for subject i is generated from $l_{i}|U_{i}=u_{i}∼\mathrm{Exp}(u_{i}\lambda )$ . We repeat the above steps and keep the subjects with $l_{i}\leq \tau_{A}$ until we get the calculated sample size n . Next we generate the waiting times between measurements for each subject according to $\mathrm{Exp}(u_{i}\lambda )$ . The number of donations for each subject is then determined by the accumulated waiting time for the donations within $\tau_{F}$ . Note that the first donation of each subject is assumed to occur at the entry time $l_{i}$ of the study so the number of donations will be added by one for each subject. The correlated binary response of subject i for the k th measurement $Y_{ik}$ is generated given the pre-specified marginal means and pairwise associations based on the method of Qaqish.^22^ The response $Y_{i1}$ is generated from Bernoulli distribution with mean $P(Y_{ik}=1|Z_{i1})$ , and response $Y_{ik}$ , $k=2,3,\ldots ,K_{i}(\tau )$ , can be generated sequentially though

$$E(Y_{ik}|{\bar{Y}}_{ik},{\bar{Z}}_{ik},Z_{ik})=E(Y_{ik}|Z_{ik})+\sum_{j=1}^{k-1}b_{ikj}(y_{ij}-E(Y_{ij}|Z_{ij})),\;k=2,3,\ldots ,K_{i}(\tau )$$

where ${\bar{Y}}_{ik}=(Y_{i1},\ldots ,Y_{i,k-1}{)}^{\prime }$ , ${\bar{Z}}_{ik}=(Z_{i1},\ldots ,Z_{i,k-1}{)}^{\prime }$ , and $b_{ikj}$ is the j th element of

$$b_{ik}({\bar{Z}}_{ik},Z_{ik})=\mathrm{cov}({\bar{Y}}_{ik}|{\bar{Z}}_{ik}{)}^{-1}\mathrm{cov}({\bar{Y}}_{ik},Y_{ik}|{\bar{Z}}_{ik},Z_{ik}),\;k=2,3,\ldots ,K_{i}(\tau )$$

Following the above simulation procedure we have the recruitment times, repeated measurement times and responses generated for all individuals in the sample. We next plan 2 interim analyses at $t_{1}$ and $t_{2}$ with a final analysis at the end of study. The average sample size required for the sequential trial under each parameter setting based on N=1000 simulations is reported in parentheses in Tables 1 and 2. The average sample size for the sequential design is slightly larger than that of a fixed study because under the current design framework—with 2 interim analyses planned at information fractions of 1/3 and 2/3—the majority of donors are recruited before the first interim analysis occurs. Nonetheless, conducting group sequential analyses remains valuable as early termination of the trial can lead to a reduction in the average sample size overall, and the average sample size remains lower than the analytically calculated sample size required for a sequential trial. The empirical rejection rates under $H_{0}$ and $H_{A}$ , respectively, based on the simulated data using the computed critical values are calculated and repeated for N=1000 times. The averaged empirical rejection rates for each scenario are summarized in Table 3 for the superiority study and in Table 4 for the non-inferiority study. From the summary tables, we see that the empirical type I errors (empirical rejection rate under $H_{0}$ ) for most of the scenarios are within 5%. Similarly for the empirical power (empirical rejection rate under $H_{A}$ ), the empirical powers reach the desired power at either 80% or 90% settings for most of the scenarios, and some cases have empirical powers slightly below the target power due to randomness. The overall empirical rejection rates are satisfying which verify our sample size calculation method for the study with interim analyses for the cluster data.

### 5.2. Empirical study with dependence between response and donation intensity

The derivations in Section 2 are based on the assumption that donations occur in the population with a conditional intensity given by (1) which does not depend on the response. Here we conduct simulation studies to examine the sensitivity of estimators to violations of this assumption in the setting where the event rate for the control arm is $\mu_{0}=0.002$ , as specified in Section 5.1. To allow the response to influence the intensity of future events, we consider the modified intensity function

(21) $$\lim_{\Delta t↓0}\frac{P(\Delta N_{i}(t)=1|H_{i}(t),U_{i}=u_{i})}{\Delta t}=u_{i}\lambda (t)\exp\;(\psi Y_{i.}(t))$$

where $Y_{i.}(t)=\sum_{k=1}^{K_{i}(t)}Y_{ik}$ is the total number of adverse events observed by study time t for subject i , i=1,…,n . According to (21), the donation intensity decreases by a factor of exp(ψ) every time an event occurs. We report simulation results for both superiority and non-inferiority trials in Table 5, where λ=10 , ϕ=0.8 , and exp(ψ)=0.5,0.9,1 , while all other parameters have the same settings as in Section 5.1. The simulations demonstrate excellent control of the type I error rate at around 5% with no appreciable loss in empirical power given that the event rate is very low. These findings suggest that when adverse events are rare, the impact of the association between adverse events and the propensity to donate is negligible on the study power. Therefore assuming independence between the donation response and donation frequency is reasonable for study planning purposes.

**Table 5.** Empirical rejection rates for a superiority trial and a non-inferiority trial with two interim analyses when the donation intensity depends on past responses through (21); λ=10 , ϕ=0.8 , R=3 , $\mu_{0}=0.002$ , $\exp\;(\beta_{1A})=1.2$ , $\tau_{A}=0.5$ , $\tau_{F}=1$ , and $\exp\;(\beta_{1M})=1.5$ .

			$\alpha_{1}^{*}(t)$				$\alpha_{2}^{*}(t)$				$\alpha_{3}^{*}(t)$			
			80% Power		90% Power		80% Power		90% Power		80% Power		90% Power	
			Emp. type	Emp.	Emp. type	Emp.	Emp. type	Emp.	Emp. type	Emp.	Emp. type	Emp.	Emp. type	Emp.
ρ	exp(ψ)	t	I error	power	I error	power	I error	power	I error	power	I error	power	I error	power
*Superiority trial*														
0.005	0.5	$t_{1}$	0.0	7.2	0.2	11.6	2.7	36.1	2.8	47.7	0.9	32.0	1.4	44.4
		$t_{2}$	2.6	46.1	2.1	54.8	1.6	28.8	1.4	31.2	2.0	29.7	1.7	30.0
		$t_{3}$	4.2	27.1	3.7	22.9	0.9	14.6	0.8	11.5	1.4	19.8	1.4	16.1
		Total	6.8	80.4	6.0	89.3	5.2	79.5	5.0	90.4	4.3	81.5	4.5	90.5
	0.9	$t_{1}$	0.0	8.0	0.1	9.2	3.1	33.4	2.4	48.5	1.7	28.0	1.9	42.0
		$t_{2}$	2.0	43.2	2.0	54.6	1.4	25.9	1.3	29.8	1.7	34.9	1.4	31.7
		$t_{3}$	4.9	28.2	3.1	25.9	1.1	17.6	0.9	12.8	1.5	17.5	1.8	16.6
		Total	6.9	79.4	5.2	89.7	5.6	76.9	4.6	91.1	4.9	80.4	5.1	90.3
	1.0	$t_{1}$	0.0	8.4	0.2	12.3	3.0	36.5	1.9	45.8	1.5	30.7	1.6	43.0
		$t_{2}$	1.9	42.9	1.9	56.0	2.0	24.8	1.2	31.6	1.5	28.3	1.1	32.9
		$t_{3}$	3.3	29.6	3.3	22.7	0.7	16.3	0.7	11.8	1.2	21.1	1.8	13.7
		Total	5.2	80.9	5.4	91.0	5.7	77.6	3.8	89.2	4.2	80.1	4.5	89.6
0.01	0.5	$t_{1}$	0.1	5.1	0.0	6.0	2.0	31.7	1.8	44.0	0.7	29.3	1.1	35.8
		$t_{2}$	2.0	41.3	2.2	57.4	1.3	29.4	1.5	31.1	1.3	29.3	1.9	35.3
		$t_{3}$	3.5	32.8	3.1	26.7	0.8	19.0	1.5	15.5	1.6	19.8	1.2	18.4
		Total	5.6	79.2	5.3	90.1	4.1	80.1	4.8	90.6	3.6	78.4	4.2	89.5
	0.9	$t_{1}$	0.0	4.4	0.0	7.4	1.7	31.1	2.6	43.7	1.0	27.7	1.1	36.1
		$t_{2}$	1.1	45.7	1.0	52.9	1.2	30.5	2.2	34.3	1.8	31.6	0.7	38.6
		$t_{3}$	3.2	31.3	2.7	29.2	1.1	19.7	1.2	13.8	0.9	20.1	2.4	15.4
		Total	4.3	81.4	3.7	89.5	4.0	81.3	6.0	91.8	3.7	79.4	4.2	90.1
	1.0	$t_{1}$	0.1	3.4	0.0	7.1	2.6	31.8	1.9	41.8	0.9	30.1	1.9	36.1
		$t_{2}$	2.1	44.5	1.2	56.0	1.5	29.1	1.7	34.2	1.6	29.4	2.8	36.2
		$t_{3}$	4.0	32.5	3.4	26.8	1.2	16.5	1.3	14.7	1.5	21.1	0.9	17.6
		Total	6.2	80.4	4.6	89.9	5.3	77.4	4.9	90.7	4.0	80.6	5.6	89.9
*Non-inferiority trial*														
0.005	0.5	$t_{1}$	0.0	6.8	0.1	10.1	2.8	38.3	3.0	48.2	1.8	32.3	2.7	45.0
		$t_{2}$	1.4	45.4	2.4	56.1	1.0	24.5	1.2	28.6	1.5	26.7	1.7	31.7
		$t_{3}$	4.0	27.9	2.8	25.1	0.6	15.4	0.9	11.6	1.4	18.8	1.3	12.6
		Total	5.4	80.1	5.3	91.3	4.4	78.2	5.1	88.4	4.7	77.8	5.7	89.3
	0.9	$t_{1}$	0.2	6.7	0.2	10.5	2.9	41.9	1.7	50.4	1.9	34.7	3.3	43.5
		$t_{2}$	0.8	42.4	1.9	53.5	1.4	26.7	1.6	27.3	1.9	29.1	1.2	29.6
		$t_{3}$	3.0	30.0	3.4	25.7	1.2	13.1	1.1	13.4	2.1	14.8	1.0	16.1
		Total	4.0	79.1	5.5	89.7	5.5	81.7	4.4	91.1	5.9	78.6	5.5	89.2
	1.0	$t_{1}$	0.0	6.4	0.0	10.7	2.8	40.9	2.0	49.6	1.1	33.7	1.5	43.8
		$t_{2}$	2.6	45.4	1.7	52.0	1.5	26.9	2.1	27.4	1.6	30.4	1.2	31.4
		$t_{3}$	3.1	27.4	2.4	25.5	0.9	14.0	1.1	12.4	1.6	16.8	1.8	14.9
		Total	5.7	79.2	4.1	88.2	5.2	81.8	5.2	89.4	4.3	80.9	4.5	90.1
0.01	0.5	$t_{1}$	0.0	3.7	0.0	7.4	2.9	33.8	2.1	45.6	1.1	33.1	1.5	40.3
		$t_{2}$	1.3	44.0	1.6	58.2	2.2	26.2	1.7	29.8	0.8	29.5	1.3	32.5
		$t_{3}$	3.9	30.9	4.1	25.1	1.0	15.9	2.0	13.7	1.6	17.2	1.9	17.0
		Total	5.2	78.6	5.7	90.7	6.1	75.9	5.8	89.1	3.5	79.8	4.7	89.8
	0.9	$t_{1}$	0.0	4.9	0.0	8.3	1.7	35.5	2.2	46.4	1.4	26.8	1.3	40.3
		$t_{2}$	1.7	41.1	1.4	54.1	1.7	27.8	1.7	29.8	1.9	30.6	1.1	34.2
		$t_{3}$	3.3	33.4	3.4	28.4	1.1	16.7	0.9	14.1	1.8	21.1	1.3	15.3
		Total	5.0	79.4	4.8	90.8	4.5	80.0	4.8	90.3	5.1	78.5	3.7	89.8
	1.0	$t_{1}$	0.2	3.6	0.0	8.0	1.2	32.7	1.6	43.4	1.8	30.3	1.4	40.6
		$t_{2}$	2.3	41.4	1.5	52.8	1.2	29.5	1.5	29.6	1.7	32.2	2.1	32.3
		$t_{3}$	2.7	33.9	4.0	28.6	1.0	15.9	1.6	14.7	1.8	17.0	1.5	14.9
		Total	5.2	78.9	5.5	89.4	3.4	78.1	4.7	87.7	5.3	79.5	5.0	87.8

### 5.3. Design of a plasma donation study

Source plasma is crucial in life-saving treatments for various medical conditions and the demand for it has consistently exceeded its supply, especially during the COVID-19 pandemic.^23^ For a potential increase in average plasma collection volume, the trial named IMPACT (IMproving PlasmA CollecTion)^24^ was conducted between 6 January and 26 March 2020 at three plasma collection centers (i.e. Charlotte, NC; Spokane, WA; and Wichita, KS) in the United States. It is a randomized controlled trial to assess the non-inferiority of a new “percent plasma nomogram” (PPN) compared to the standard nomogram in terms of the incidence of significant hypotensive adverse events.^24^ The PPN nomogram was developed to personalize the target plasma volume for each donor, allowing for adjustment of the target plasma volume upward or downward based on individual characteristics. Donors were recruited between 6 January and 26 March 2020 at three plasma collection centers: Charlotte, NC; Spokane, WA; and Wichita, KS. Here we demonstrate how the proposed design framework can be applied to plan a plasma donation study with interim analyses in this context.

To design the study based on the aims of the IMPACT trial, we assume $P(X_{i}=1)=0.5$ so that donors are randomized into two groups with a 50:50 allocation to either the PPN or the standard nomogram arm. Based on Canadian plasma donor data on the expected annual number of donations per donor is ∼ 21 so we set λ=21 . To account for variability in donation frequency among donors we set ϕ=0.80 . The rate of significant hypotensive adverse events in the control arm using the standard nomogram is set to $\mu_{0}=0.035\%$ . For the experimental arm, we anticipate a 50% increase in the event rate, so we let $\exp\;(\beta_{1A})=1.50$ . Given the low event rate we set a non-inferiority margin of $\exp\;(\beta_{1M})=2.5$ , and consider trial planning with $H_{0}:\beta_{1}\geq \beta_{1M}$ versus $H_{A}:\beta_{1}<\beta_{1M}$ with significant level α=5% and target power at 80%. We assume an exchangeable within-donor serial correlation of ρ=0.005 across repeated donations. Furthermore, we plan for a 6-month recruitment duration ( $\tau_{A}=0.5$ ) and follow each donor for 6 months ( $\tau_{F}=0.5$ ).

**Figure 2.**
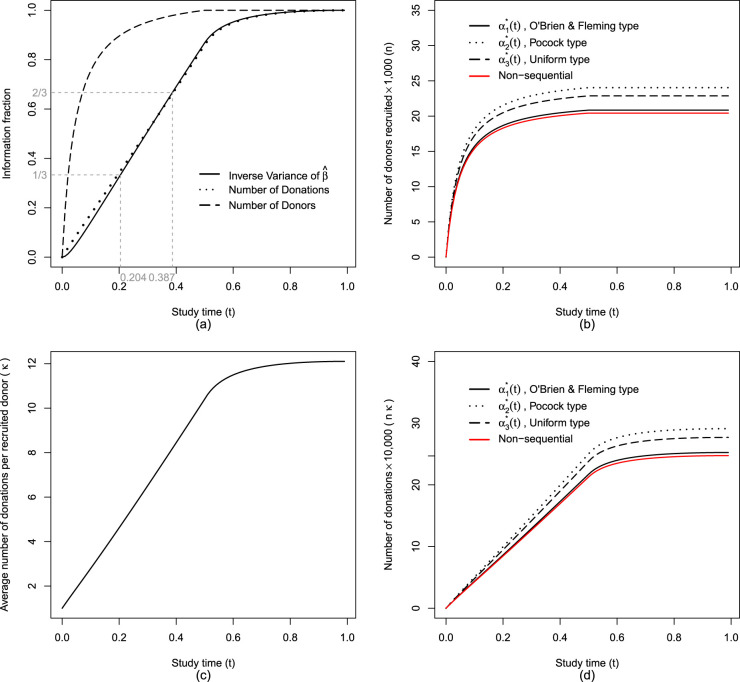
Study progression over time in a non-inferiority trial of information fraction, number of donors recruited, average donations per donor, and total donations with λ=21 , ϕ=0.8 , $\mu_{0}=0.035\%$ , $\exp\;(\beta_{1A})=1.5$ , $\exp\;(\beta_{1M})=2.5$ , ρ=0.005 , $\tau_{F}=0.5$ , α=5% , and target power=80%.

We plan to conduct two interim analyses at times $t_{1}$ and $t_{2}$ , along with a final analysis at the end of the study, $t_{3}=\tau_{A}+\tau_{F}=1$ . The timing of the interim analyses can be determined by the amount of information accumulated up to each point in the study. Figure 2(a) illustrates the growth of the information fraction over the course of the study based on the inverse variance of the estimator ${\hat{\beta }}_{1}$ . For example, interim analyses can be conducted at study times approximately $t_{1}=0.20$ and $t_{2}=0.40$ which corresponds to 1/3 and 2/3 of the maximum information expected by the end of the study. If the estimated variance of ${\hat{\beta }}_{1}$ is unavailable, alternative metrics such as the proportion of donors recruited, donations accumulated, or simply the passage of study time can be used to approximate the information fraction and guide the timing of interim analyses. The critical values for three analyses, that is, first interim analysis at $t_{1}$ , second interim analysis at $t_{2}$ , and final analysis at $t_{3}=1$ , are provided in Table D1 in the Supplemental Material. These critical values are calculated based on the information fraction defined as the fraction of inverse variance of ${\hat{\beta }}_{1}$ . Here for a fixed design the critical value is around 1.64 for a one-sided test with significant level α=5% . Using an O’Brien and Fleming alpha spending function $\alpha_{1}^{*}(t)$ , the critical values at $t_{1}=0.2$ , $t_{2}=0.4$ , and $t_{3}=1$ are 3.25, 2.09, and 1.70, respectively. For a Pocock-type spending function $\alpha_{2}^{*}(t)$ , the corresponding critical values are 2.01, 1.96, and 1.98, while for a uniform spending function $\alpha_{3}^{*}(t)$ they are 2.14, 1.96, and 1.88; see Table D1 in the Supplemental Material. The O’Brien-Fleming alpha spending function heavily protects against early stopping by setting a very strict (high) critical value for the first interim analysis with more relaxed critical values toward the final analysis. Pocock type alpha spending function provides more balanced critical values across all interim analyses, potentially allowing for earlier termination of the trial since the first interim analysis has a considerably lower critical value compared to the O’Brien-Fleming type. The uniform alpha spending function allocates the type I error rate uniformly across all interim analyses and it falls between O’Brien-Fleming and Pocock in terms of conservatism on early stopping.

Figure 2(b) shows the number of donors recruited over time. Under the same setting a non-sequential study requires the fewest donors ( n=20,426 ) to achieve 80% power and a study planned using Pocock type alpha spending function requires the largest number of donors ( n=24,031 ). If the study is terminated early following interim analyses, the total number of recruited donors will be fewer than the sample size originally planned for the end of the study. The average number of donations per recruited donor (Figure 2(c)) and number of donations (Figure 2(d)) increase rapidly during the recruitment period as the frequent donors are encountered and recruited earlier.

## 6. Discussion

We have developed a framework for the design and analysis of large scale trials involving marked point processes where there is inter-individual variation in the rate of events. The events are the occasions in which a therapeutic intervention is warranted and the trial is planned such that the same intervention is applied each time an event occurs during the course of follow-up. We consider the setting where the response is available relatively soon after the therapy is administered and it does not affect the intensity for future events. This is suitable for the study of chronic conditions with recurrent episodic flares or acute exacerbations that warrant treatment and in the introduction we listed example settings.

The methodological development here is specifically motivated by challenges in blood donation research where the goal, like that of Hartmann et al.^24^ is to show that a new device or algorithm is used to extract blood in donors is safe. For this specific setting, we use a binary response indicating the occurrence of a serious hypotensive adverse event wherein the probability of such an event is very rare—this, along with the desire to estimate the relative rate of events, leads us to the use of a log link function. A working independence assumption means the dependence structure does not need to be specified but protection against misspecification of the variance function (implicit from the use of the Poisson approximation and log link) and serial dependence in responses is ensured by use of a robust covariance matrix. While we formulate our method based on a binary response, other types of responses could be considered provided they are measurable soon after the donation.

The problem we address has similarities with the problem of group sequential monitoring of cluster-randomized trials with variable cluster sizes (e.g. Grayling et al.^25^), but it differs in several key respects. First in our setting clusters are defined by individuals, and the number of donations within an individual arises from a point process, rather than the way individuals might be organized in static clusters. Second, we deal with the correlation of responses over successive donations using marginal models for correlated binary data rather than mixed effect modeling. Third, heterogeneity in the donation rate in the target population can be characterized using historical data in the donation setting and it represents a meaningful population parameter (e.g. the variance of the random effect in the mixed Poisson process) when modeling the donation process. This heterogeneity helps trialists anticipate that the donation rate among donors recruited earlier during the accrual period will tend to be higher than those recruited towards the end of the accrual period. A consequence of this phenomenon is that the rate of information acquisition is very much higher early in the course of the study than a later phase when the highest frequency of donors were already recruited and may have completed their follow-up—less frequent donors being followed at this point would be yielding less information per unit of calendar time. The derivations of the joint distributions of successive test statistics and specification of alpha spending functions are exploited here to ensure interim analyses and stopping rules are possible while ensuring control of the type I error rate. This has significant implications on the optimal timing of interim analyses, and trials aiming to plan analyses at roughly equal increments of information must ensure that data collection, validation and reporting is possible soon after the launch of the trial to ensure that safety can be effectively monitored.

The derivations we provide can also be exploited when interest lies in conditional power^26^ calculations. If there is a concern that sample size calculations were inadequate due to misspecification of one or more parameters (e.g. the baseline event rate determined by $\beta_{0}$ ) then the joint distribution of the current and a future test statistic can be useful to calculate the probability that the trial at the planned or some later end of study will lead to rejection of the null hypothesis. This may be done in the context of a fixed sample trial or a sequential trial, and can be used as a basis for sample size re-estimation^27^ or stochastic curtailment^28^ of a trial.

## Supplemental Material

sj-pdf-1-smm-10.1177_09622802251350263 - Supplemental material for Group sequential analysis of marked point processes: Plasma donation trials Supplemental material, sj-pdf-1-smm-10.1177_09622802251350263 for Group sequential analysis of marked point processes: Plasma donation trials by Kecheng Li and Richard J Cook in Statistical Methods in Medical Research
