# Supplementary material for: Group sequential analysis of marked point processes: Plasma donation trials
Source: Stat Methods Med Res. 2025 Jul 2;34(8):1646–64. doi: 10.1177/09622802251350263 (PMC12365355; doi:10.1177/09622802251350263)
Supplement: sj-pdf-1-smm-10.1177_09622802251350263 - Supplemental material for Group sequential analysis of marked point processes: Plasma donation trials [file sj-pdf-1-smm-10.1177_09622802251350263.pdf]

Supplementary Material for Group Sequential Analysis of Marked Point Processes: Plasma Donation Trials

A | DERIVATIONS OF THE MATRICES $\mathcal{A}(t_r)$, $\mathcal{B}(t_r)$ AND $\mathcal{B}(t_r, t_s)$ IN A SIMPLIFIED TWO-ARM TRIAL

If we constrain $\boldsymbol{\beta}_2 = \mathbf{0}$ (i.e. without the adjustment for covariates other than the assigned treatment), $\mathbf{Z}_{ik} = \mathbf{Z}_i = (1, X_i)'$ for $k = 1, \ldots, K_i(t_r)$, and $\boldsymbol{\beta} = (\beta_1, \beta_0)'$. Hence the means of responses are the same over repeated measurements, i.e. $\mu(\mathbf{Z}_{ik}; \boldsymbol{\beta}) = \mu(X_i; \boldsymbol{\beta})$. If we let $\mu(X_i) = \mu(X_i; \boldsymbol{\beta})$ for simplification, and specifically with the log link $D_i(t_r; \boldsymbol{\beta})$ is a $K_i(t_r) \times 2$ matrix of the form

$$D_i(t_r; \boldsymbol{\beta}) = \frac{\partial \boldsymbol{\mu}_i(t_r; \boldsymbol{\beta})}{\partial \boldsymbol{\beta}'} = \mu(X_i) \begin{pmatrix} X_i & 1 \\ \vdots & \vdots \\ X_i & 1 \end{pmatrix} . \tag{A.1}$$

With the identity (Poisson) variance function and a working independence assumption, the $K_i(t_r) \times K_i(t_r)$ working covariance matrix has identical entries in the diagonal,

$$\Sigma_i^{-1}(t_r; \boldsymbol{\beta}, \boldsymbol{\alpha}) = \Sigma_i^{-1}(t_r; \boldsymbol{\beta}) = \begin{pmatrix} \mu^{-1}(X_i) & & 0 \\ & \ddots & \\ 0 & & \mu^{-1}(X_i) \end{pmatrix} . \tag{A.2}$$

The generalized estimating function in (2) then simplifies to

$$\begin{aligned} \boldsymbol{U}_i(t_r; \boldsymbol{\beta}) =& \Delta_i(t_r)\mu(X_i) \begin{pmatrix} X_i & \cdots & X_i \\ 1 & \cdots & 1 \end{pmatrix} \begin{pmatrix} \mu^{-1}(X_i) & & 0 \\ & \ddots & \\ 0 & & \mu^{-1}(X_i) \end{pmatrix} \begin{pmatrix} Y_{i1} - \mu(X_i) \\ \vdots \\ Y_{i,K_i(t_r)} - \mu(X_i) \end{pmatrix} \\ =& \Delta_i(t_r) \begin{pmatrix} X_i & \cdots & X_i \\ 1 & \cdots & 1 \end{pmatrix} \begin{pmatrix} Y_{i1} - \mu(X_i) \\ \vdots \\ Y_{i,K_i(t_r)} - \mu(X_i) \end{pmatrix} \\ =& \Delta_i(t_r) \begin{pmatrix} \sum_{j=1}^{K_i(t_r)} X_i(Y_{ij} - \mu(X_i)) \\ \sum_{j=1}^{K_i(t_r)} (Y_{ij} - \mu(X_i)) \end{pmatrix} \\ =& \Delta_i(t_r) \sum_{j=1}^{K_i(t_r)} \begin{pmatrix} U_{ij1}(\beta) \\ U_{ij2}(\beta) \end{pmatrix} . \end{aligned}$$

A.1 | Derivation of the $\mathcal{A}(t_r)$ Matrix

The $\mathcal{A}(t_r)$ is given more explicitly by

$$\begin{aligned}
\mathcal{A}(t_r) &= \mathbb{E}\left\{\Delta_i(t_r) D_i'(t_r;\boldsymbol{\beta})\,\Sigma_i^{-1}(t_r;\boldsymbol{\beta},\boldsymbol{\alpha})\,D_i(t_r;\boldsymbol{\beta})\right\} \\
&= \mathbb{E}\left\{\Delta_i(t_r)\,\mu(X_i)\begin{pmatrix} X_i & \cdots & X_i \\ 1 & \cdots & 1\end{pmatrix}\begin{pmatrix}\mu^{-1}(X_i) & & 0 \\ & \ddots & \\ 0 & & \mu^{-1}(X_i)\end{pmatrix}\mu(X_i)\begin{pmatrix} X_i & 1 \\ \vdots & \vdots \\ X_i & 1\end{pmatrix}\right\} \\
&= \mathbb{E}\left\{\Delta_i(t_r)\begin{pmatrix} X_i & \cdots & X_i \\ 1 & \cdots & 1\end{pmatrix}\mu(X_i)\begin{pmatrix} X_i & 1 \\ \vdots & \vdots \\ X_i & 1\end{pmatrix}\right\} \\
&= \mathbb{E}\left\{\Delta_i(t_r)\begin{pmatrix} K_i(t_r)X_i\,\mu(X_i) & K_i(t_r)X_i\,\mu(X_i) \\ K_i(t_r)X_i\,\mu(X_i) & K_i(t_r)\,\mu(X_i)\end{pmatrix}\right\}.
\end{aligned}$$

Let $\kappa_x(t_r) = \mathbb{E}\{K_i(t_r) \mid X_i = x, \Delta_i(t_r) = 1\}$ be the expected number of measurements for a subject in group x for the rth interim analysis. Let $K_i(t_r, t_s)$ be the number of transfusions needed over time period $(t_r, t_s]$ for individual i, then $\kappa_x(t_r, t_s) = \mathbb{E}\{K_i(t_r, t_s) \mid X_i = x, \Delta_i(t_r) = 1\}$ is the expected number of measurements from study time t_r to t_s for a subject i in group x, and $\nu_x(t_r) = \mathrm{var}(K_i(t_r) \mid X_i = x, \Delta_i(t_r) = 1)$ be the variance of these measurement counts, $x = 0, 1$. Let $\mu_x = \mu(X_i = x)$ and let $V_x = \mathrm{var}(Y_{ij} \mid X_i = x)$ be the true variance of responses for the subject in group x so that $V_x = \mu_x(1-\mu_x)$, $x = 0, 1$. Additionally let $\pi(t_r)$ denote $P(\Delta_i(t_r) = 1)$, then further taking the expectation with respect to $K_i(t_r)$, X_i and $\Delta_i(t_r)$ we obtain

$$\mathcal{A}(t_r) = \pi(t_r)\begin{pmatrix} \kappa_1(t_r)\,\mu_1 P(X_i = 1) & \kappa_1(t_r)\,\mu_1 P(X_i = 1) \\ \kappa_1(t_r)\,\mu_1 P(X_i = 1) & \sum_{x=0}^{1}\kappa_x(t_r)\,\mu_x P(X_i = x)\end{pmatrix}. \tag{A.3}$$

A.2 | Derivation of the $\mathcal{B}(t_r)$ Matrix

We have

$$\begin{aligned}
\mathcal{B}(t_r) &= \mathbb{E}\{\boldsymbol{U}_i(t_r;\boldsymbol{\beta})\boldsymbol{U}_i'(t_r;\boldsymbol{\beta})\} \\
&= \mathbb{E}\left\{\begin{matrix} \Delta_i(t_r)\sum_{j=1}^{K_i(t_r)}\sum_{k=1}^{K_i(t_r)} U_{ij1}(\beta)U_{ik1}(\beta) & \Delta_i(t_r)\sum_{j=1}^{K_i(t_r)}\sum_{k=1}^{K_i(t_r)} U_{ij1}(\beta)U_{ik2}(\beta) \\ \Delta_i(t_r)\sum_{j=1}^{K_i(t_r)}\sum_{k=1}^{K_i(t_r)} U_{ij1}(\beta)U_{ik2}(\beta) & \Delta_i(t_r)\sum_{j=1}^{K_i(t_r)}\sum_{k=1}^{K_i(t_r)} U_{ij2}(\beta)U_{ik2}(\beta)\end{matrix}\right\}.
\end{aligned} \tag{A.4}$$

For the (1,1) entry of (A.4) we have

$$\mathbb{E}\left\{\Delta_i(t_r)\sum_{j=1}^{K_i(t_r)}\sum_{k=1}^{K_i(t_r)} U_{ij1}(\beta)\, U_{ik1}(\beta) \,\middle|\, X_i, K_i(t_r), \Delta_i(t_r)\right\}$$

$$= \Delta_i(t_r)\mathbb{E}\left\{\sum_{j=1}^{K_i(t_r)}\sum_{k=1}^{K_i(t_r)}(Y_{ij}-\mu(X_i))\, X_i\, (Y_{ik}-\mu(X_i)) \,\middle|\, X_i, K_i(t_r), \Delta_i(t_r)\right\}$$

$$= \Delta_i(t_r)\mathbb{E}\left\{\sum_{j=1}^{K_i(t_r)} X_i U_{ij2}^2 + \sum_{\substack{j=1\\ j\neq k}}^{K_i(t_r)}\sum_{k=1}^{K_i(t_r)} X_i\,(Y_{ij}-\mu(X_i))\,(Y_{ik}-\mu(X_i)) \,\middle|\, X_i, K_i(t_r), \Delta_i(t_r)\right\}$$

$$= \Delta_i(t_r)\left\{K_i(t_r)\,\mathrm{var}(Y_{ij} \mid X_i = 1) + K_i(t_r)(K_i(t_r)-1)\,\mathrm{cov}(Y_{ij}, Y_{ik} \mid X_i = 1)\right\} I(X_i = 1)\,.$$

Taking the expectation with respect to $K_i(t_r)$, X_i and $\Delta_i(t_r)$ gives

$$\pi(t_r)\left[\kappa_1(t_r)\,\mathrm{var}(Y_{ij} \mid X_i = 1) + \{\nu_1(t_r) + \kappa_1^2(t_r) - \kappa_1(t_r)\}\,\mathrm{cov}(Y_{ij}, Y_{ik} \mid X_i = 1)\right] P(X_i = 1)\,.$$

For the (1,2) entry given X_i, $K_i(t_r)$ and $\Delta_i(t_r)$ we get

$$\Delta_i(t_r)\left\{K_i(t_r)\,\mathrm{var}(Y_{ij} \mid X_i = 1) + K_i(t_r)(K_i(t_r)-1)\,\mathrm{cov}(Y_{ij}, Y_{ik} \mid X_i = 1)\right\} I(X_i = 1)\,,$$

and overall we have

$$\pi(t_r)\left[\kappa_1(t_r)\,\mathrm{var}(Y_{ij} \mid X_i = 1) + \{\nu_1(t_r) + \kappa_1^2(t_r) - \kappa_1(t_r)\}\,\mathrm{cov}(Y_{ij}, Y_{ik} \mid X_i = 1)\right] P(X_i = 1)\,.$$

Regarding the (2, 2) entry of (A.4) note that

$$\mathbb{E}\left\{\Delta_i(t_r)\sum_{j=1}^{K_i(t_r)}\sum_{k=1}^{K_i(t_r)} U_{ij2}(\beta) U_{ik2}(\beta) \,\middle|\, X_i, K_i(t_r), \Delta_i(t_r)\right\}$$

$$= \Delta_i(t_r)\mathbb{E}\left\{\sum_{j=1}^{K_i(t_r)} U_{ij2}^2(\beta) + \sum_{\substack{j=1\\ j\neq k}}^{K_i(t_r)}\sum_{k=1}^{K_i(t_r)} U_{ij2}(\beta) U_{ik2}(\beta) \,\middle|\, X_i, K_i(t_r), \Delta_i(t_r)\right\}$$

$$= \Delta_i(t_r)\left\{K_i(t_r)\,\mathrm{var}(Y_{ij} \mid X_i) + \sum_{\substack{j=1\\ j\neq k}}^{K_i(t_r)}\sum_{k=1}^{K_i(t_r)} \mathrm{cov}(Y_{ij}, Y_{ik} \mid X_i)\right\}$$

$$= \Delta_i(t_r)\left\{K_i(t_r)\,\mathrm{var}(Y_{ij} \mid X_i) + K_i(t_r)\,(K_i(t_r)-1)\,\mathrm{cov}(Y_{ij}, Y_{ik} \mid X_i)\right\}\,. \tag{A.5}$$

This has expectation

$$\sum_{x=0}^{1}\left[\mathbb{E}\{K_i(t_r) \mid X_i = x\}\,\mathrm{var}(Y_{ij} \mid X_i = x)\, P(X_i = x) + \mathbb{E}\{K_i(t_r)(K_i(t_r)-1) \mid X_i = x\}\,\mathrm{cov}(Y_{ij}, Y_{ik} \mid X_i = x)\, P(X_i = x)\right] P(\Delta_i(t_r) = 1)$$

$$= \pi(t_r)\sum_{x=0}^{1}\left[\kappa_x(t_r)\,\mathrm{var}(Y_{ij} \mid X_i = x) + \{\nu_x(t_r) + \kappa_x^2(t_r) - \kappa_x(t_r)\}\,\mathrm{cov}(Y_{ij}, Y_{ik} \mid X_i = x)\right] P(X_i = x)\,.$$

If we let $\mathrm{corr}(Y_{ij}, Y_{ik} \mid X_i = x) = \rho$ be an exchangeable correlation of responses within individuals we can express $\mathrm{cov}(Y_{ij}, Y_{ik} \mid X_i = x)$ as

$$\mathrm{cov}(Y_{ij}, Y_{ik} \mid X_i = x) = \rho \cdot \mathrm{var}(Y_{ij} \mid X_i = x)\,.$$

Hence

$$\mathcal{B}(t_r) = \pi(t_r) \begin{pmatrix} P(X_i = 1)\left\{\kappa_1(t_r)V_1 + \delta_1(t_r)\rho V_1\right\} & P(X_i = 1)\left\{\kappa_1(t_r)V_1 + \delta_1(t_r)\rho V_1\right\} \\ P(X_i = 1)\left\{\kappa_1(t_r)V_1 + \delta_1(t_r)\rho V_1\right\} & \sum_{x=0}^{1} P(X_i = x)\left\{\kappa_x(t_r)V_x + \delta_x(t_r)\rho V_x\right\} \end{pmatrix}, \tag{A.6}$$

where $\delta_x(t_r) = \nu_x(t_r) + \kappa_x^2(t_r) - \kappa_x(t_r)$.

A.3 | Derivation of the Covariance Matrix $\mathcal{B}(t_r, t_s)$

Recall that $\boldsymbol{U}_i(t_r; \boldsymbol{\beta})$ is given by

$$\boldsymbol{U}_i(t_r; \boldsymbol{\beta}) = \Delta_i(t_r) \begin{pmatrix} \sum_{j=1}^{K_i(t_r)} X_i(Y_{ij} - \mu(X_i)) \\ \sum_{j=1}^{K_i(t_r)} (Y_{ij} - \mu(X_i)) \end{pmatrix} = \Delta_i(t_r) \sum_{j=1}^{K_i(t_r)} \begin{pmatrix} U_{ij1}(\beta) \\ U_{ij2}(\beta) \end{pmatrix}.$$

Suppose $t_r < t_s$ so we have $K_i(t_r) \leq K_i(t_s)$ and

$$\begin{aligned} \mathcal{B}(t_r, t_s) &= \mathbb{E}\{\boldsymbol{U}_i(t_r; \boldsymbol{\beta})\boldsymbol{U}_i'(t_s; \boldsymbol{\beta})\} \\ &= \mathbb{E}\left\{ \begin{matrix} \Delta_i(t_r) \sum_{j=1}^{K_i(t_r)} \sum_{k=1}^{K_i(t_s)} U_{ij1}(\beta)U_{ik1}(\beta) & \Delta_i(t_r) \sum_{j=1}^{K_i(t_r)} \sum_{k=1}^{K_i(t_s)} U_{ij1}(\beta)U_{ik2}(\beta) \\ \Delta_i(t_r) \sum_{j=1}^{K_i(t_r)} \sum_{k=1}^{K_i(t_s)} U_{ij1}(\beta)U_{ik2}(\beta) & \Delta_i(t_r) \sum_{j=1}^{K_i(t_r)} \sum_{k=1}^{K_i(t_s)} U_{ij2}(\beta)U_{ik2}(\beta) \end{matrix} \right\}. \end{aligned} \tag{A.7}$$

For the (1,1) entry of (A.7) we have

$$\begin{aligned} &\mathbb{E}\left\{ \Delta_i(t_r) \sum_{j=1}^{K_i(t_r)} \sum_{k=1}^{K_i(t_s)} U_{ij1}(\beta)\, U_{ik1}(\beta) \,\middle|\, X_i, K_i(t_r), K_i(t_s), \Delta_i(t_r) \right\} \\ &= \Delta_i(t_r)\mathbb{E}\left\{ \sum_{j=1}^{K_i(t_r)} \sum_{k=1}^{K_i(t_s)} (Y_{ij} - \mu(X_i))\, X_i\, (Y_{ik} - \mu(X_i)) \,\middle|\, X_i, K_i(t_r), K_i(t_s), \Delta_i(t_r) \right\} \\ &= \Delta_i(t_r)\mathbb{E}\Bigg\{ \sum_{j=1}^{K_i(t_r)} X_i U_{ij2}^2 + \sum_{j=1}^{K_i(t_r)} \sum_{\substack{k=1 \\ j \neq k}}^{K_i(t_r)} X_i\, (Y_{ij} - \mu(X_i))\, (Y_{ik} - \mu(X_i)) \\ &\quad + I(K_i(t_s) > K_i(t_r)) \sum_{j=1}^{K_i(t_r)} \sum_{k=K_i(t_r)+1}^{K_i(t_s)} X_i\, (Y_{ij} - \mu(X_i))\, (Y_{ik} - \mu(X_i)) \,\Bigg|\, X_i, K_i(t_r), K_i(t_s), \Delta_i(t_r) \Bigg\} \\ &= \Delta_i(t_r)\big\{ K_i(t_r)\operatorname{var}(Y_{ij} \mid X_i = 1) + K_i(t_r)(K_i(t_r) - 1)\operatorname{cov}(Y_{ij}, Y_{ik} \mid X_i = 1) + K_i(t_r)(K_i(t_s) - K_i(t_r))\operatorname{cov}(Y_{ij}, Y_{ik} \mid X_i = 1) \big\}\, I(X_i = 1) \\ &= \Delta_i(t_r)\left\{ K_i(t_r)\operatorname{var}(Y_{ij} \mid X_i = 1) + K_i(t_r)(K_i(t_s) - 1)\operatorname{cov}(Y_{ij}, Y_{ik} \mid X_i = 1) \right\}\, I(X_i = 1)\,. \end{aligned}$$

Taking the expectation with respect to $K_i(t_r)$, $K_i(t_s)$, X_i and $\Delta_i(t_r)$ gives

$$\pi(t_r)\left[\kappa_1(t_r)\operatorname{var}(Y_{ij} \mid X_i = 1) + \{\nu_1(t_r) + \kappa_1^2(t_r) + \kappa_1(t_r)\kappa_1(t_r, t_s) - \kappa_1(t_r)\}\operatorname{cov}(Y_{ij}, Y_{ik} \mid X_i = 1) \right] P(X_i = 1)\,.$$

For the (1,2) entry of (A.7) given X_i, $K_i(t_r)$, $K_i(t_s)$ and $\Delta_i(t_r)$ we get

$$\Delta_i(t_r)\left\{K_i(t_r)\,\mathrm{var}(Y_{ij}\mid X_i=1)+K_i(t_r)(K_i(t_s)-1)\,\mathrm{cov}(Y_{ij},Y_{ik}\mid X_i=1)\right\}\,I(X_i=1)\,,$$

and overall we have

$$\pi(t_r)\left[\kappa_1(t_r)\,\mathrm{var}(Y_{ij}\mid X_i=1)+\{\nu_1(t_r)+\kappa_1^2(t_r)+\kappa_1(t_r)\kappa_1(t_r,t_s)-\kappa_1(t_r)\}\,\mathrm{cov}(Y_{ij},Y_{ik}\mid X_i=1)\right]P(X_i=1)\,.$$

Regarding the (2, 2) entry of (A.7) note that

$$\begin{aligned}
&\mathbb{E}\left\{\Delta_i(t_r)\sum_{j=1}^{K_i(t_r)}\sum_{k=1}^{K_i(t_s)}U_{ij2}(\beta)U_{ik2}(\beta)\,\middle|\,X_i,K_i(t_r),K_i(t_s),\Delta_i(t_r)\right\} \qquad (A.8)\\
=&\Delta_i(t_r)\mathbb{E}\left\{\sum_{j=1}^{K_i(t_r)}U_{ij2}^2(\beta)+\sum_{\substack{j=1\\ j\neq k}}^{K_i(t_r)}\sum_{k=1}^{K_i(t_s)}U_{ij2}(\beta)U_{ik2}(\beta)\,\middle|\,X_i,K_i(t_r),K_i(t_s),\Delta_i(t_r)\right\}\\
=&\Delta_i(t_r)\left\{K_i(t_r)\,\mathrm{var}(Y_{ij}\mid X_i)+\sum_{\substack{j=1\\ j\neq k}}^{K_i(t_r)}\sum_{k=1}^{K_i(t_s)}\mathrm{cov}(Y_{ij},Y_{ik}\mid X_i)\right\}\\
=&\Delta_i(t_r)\left\{K_i(t_r)\,\mathrm{var}(Y_{ij}\mid X_i)+\sum_{\substack{j=1\\ j\neq k}}^{K_i(t_r)}\sum_{k=1}^{K_i(t_r)}\mathrm{cov}(Y_{ij},Y_{ik}\mid X_i)+I(K_i(t_s)>K_i(t_r))\sum_{j=1}^{K_i(t_r)}\sum_{k=K_i(t_r)+1}^{K_i(t_s)}\mathrm{cov}(Y_{ij},Y_{ik}\mid X_i)\right\}\\
=&\Delta_i(t_r)\left\{K_i(t_r)\,\mathrm{var}(Y_{ij}\mid X_i)+K_i(t_r)(K_i(t_r)-1)\mathrm{cov}(Y_{ij},Y_{ik}\mid X_i)+K_i(t_r)(K_i(t_s)-K_i(t_r))\mathrm{cov}(Y_{ij},Y_{ik}\mid X_i)\right\}\\
=&\Delta_i(t_r)\left\{K_i(t_r)\,\mathrm{var}(Y_{ij}\mid X_i)+K_i(t_r)\,(K_i(t_s)-1)\,\mathrm{cov}(Y_{ij},Y_{ik}\mid X_i)\right\}\;.
\end{aligned}$$

This has expectation

$$\begin{aligned}
&\sum_{x=0}^{1}\left[\mathbb{E}\{K_i(t_r)\mid X_i=x\}\,\mathrm{var}(Y_{ij}\mid X_i=x)\,P(X_i=x)+\mathbb{E}\{K_i(t_r)(K_i(t_s)-1)\mid X_i=x\}\,\mathrm{cov}(Y_{ij},Y_{ik}\mid X_i=x)\,P(X_i=x)\right]P(\Delta_i(t_r)=1)\\
&=\pi(t_r)\sum_{x=0}^{1}\left[\kappa_x(t_r)\,\mathrm{var}(Y_{ij}\mid X_i=x)+\{\nu_x(t_r)+\kappa_x^2(t_r)+\kappa_x(t_r)\kappa_x(t_r,t_s)-\kappa_x(t_r)\}\,\mathrm{cov}(Y_{ij},Y_{ik}\mid X_i=x)\right]P(X_i=x)\,.
\end{aligned}$$

Hence

$$\mathcal{B}(t_r,t_s)=\pi(t_r)\begin{pmatrix} P(X_i=1)\left\{\kappa_1(t_r)V_1+\delta_1(t_r,t_s)\rho V_1\right\} & P(X_i=1)\left\{\kappa_1(t_r)V_1+\delta_1(t_r,t_s)\rho V_1\right\}\\ P(X_i=1)\left\{\kappa_1(t_r)V_1+\delta_1(t_r,t_s)\rho V_1\right\} & \sum_{x=0}^{1}P(X_i=x)\left\{\kappa_x(t_r)V_x+\delta_x(t_r,t_s)\rho V_x\right\}\end{pmatrix}\,, \qquad (A.9)$$

where $\delta_x(t_r,t_s)=\nu_x(t_r)+\kappa_x^2(t_r)+\kappa_x(t_r)\kappa_x(t_r,t_s)-\kappa_x(t_r)$.

B | DERIVATIONS FOR A TWO-ARM CLINICAL TRIAL

B.1 | Simplification of the Asymptotic Variance of $\widehat{\beta}_1(t_r)$

With 50:50 randomization we can write (A.3) as

$$\mathcal{A}(t_r)=\pi(t_r)\begin{pmatrix}\dfrac{\kappa_1(t_r)\mu_1}{2} & \dfrac{\kappa_1(t_r)\mu_1}{2}\\ \dfrac{\kappa_1(t_r)\mu_1}{2} & \dfrac{\kappa_0(t_r)\mu_0}{2}+\dfrac{\kappa_1(t_r)\mu_1}{2}\end{pmatrix}\,,$$

and (A.6) as

$$\mathcal{B}(t_r) = \pi(t_r) \begin{pmatrix} \frac{1}{2}\{\kappa_1(t_r)V_1 + \delta_1(t_r)\rho V_1\} & \frac{1}{2}\{\kappa_1(t_r)V_1 + \delta_1(t_r)\rho V_1\} \\ \frac{1}{2}\{\kappa_1(t_r)V_1 + \delta_1(t_r)\rho V_1\} & \frac{1}{2}\{\kappa_0(t_r)V_0 + \delta_0(t_r)\rho V_0\} + \frac{1}{2}\{\kappa_1(t_r)V_1 + \delta_1(t_r)\rho V_1\} \end{pmatrix},$$

and (A.9) as

$$\mathcal{B}(t_r, t_s) = \pi(t_r) \begin{pmatrix} \frac{1}{2}\{\kappa_1(t_r)V_1 + \delta_1(t_r, t_s)\rho V_1\} & \frac{1}{2}\{\kappa_1(t_r)V_1 + \delta_1(t_r, t_s)\rho V_1\} \\ \frac{1}{2}\{\kappa_1(t_r)V_1 + \delta_1(t_r, t_s)\rho V_1\} & \frac{1}{2}\{\kappa_0(t_r)V_0 + \delta_0(t_r, t_s)\rho V_0\} + \frac{1}{2}\{\kappa_1(t_r)V_1 + \delta_1(t_r, t_s)\rho V_1\} \end{pmatrix}.$$

Recall that

$$\begin{aligned} M_2(t_r, t_s) &= \mathrm{cov}(\bar{U}_i(t_r; \beta_1, \boldsymbol{\theta}, \boldsymbol{\alpha}), \bar{U}_i(t_s; \beta_1, \boldsymbol{\theta}, \boldsymbol{\alpha})) \\ &= \mathcal{B}_{\beta_1\beta_1}(t_r, t_s) + \mathcal{A}_{\beta_1\boldsymbol{\theta}}(t_r)\mathcal{A}^{-1}_{\boldsymbol{\theta\theta}}(t_r)\mathcal{B}_{\boldsymbol{\theta\theta}}(t_r, t_s)[\mathcal{A}^{-1}_{\boldsymbol{\theta\theta}}(t_s)]'[\mathcal{A}_{\beta_1\boldsymbol{\theta}}(t_s)]' \\ &\quad - \mathcal{B}_{\beta_1\boldsymbol{\theta}}(t_r, t_s)[\mathcal{A}^{-1}_{\boldsymbol{\theta\theta}}(t_s)]'[\mathcal{A}_{\beta_1\boldsymbol{\theta}}(t_s)]' - \mathcal{A}_{\beta_1\boldsymbol{\theta}}(t_r)\mathcal{A}^{-1}_{\boldsymbol{\theta\theta}}(t_r)\mathcal{B}_{\boldsymbol{\theta}\beta_1}(t_r, t_s). \end{aligned}$$

Then letting $a_x(t_r, t_s) = \frac{1}{2}\{\kappa_x(t_r)V_x + \delta_x(t_r, t_s)\rho V_x\}$, $b_x(t_r) = \kappa_x(t_r)\mu_x/2$, $x = 0, 1$,

$$\begin{aligned} M_2(t_r, t_s) &= \pi(t_r)\Big\{a_1(t_r, t_s) + \frac{b_1(t_r)}{b_0(t_r) + b_1(t_r)}(a_0(t_r, t_s) + a_1(t_r, t_s))\frac{b_1(t_s)}{b_0(t_s) + b_1(t_s)} \\ &\quad - \frac{a_1(t_r, t_s)b_1(t_s)}{b_0(t_s) + b_1(t_s)} - \frac{a_1(t_r, t_s)b_1(t_r)}{b_0(t_r) + b_1(t_r)}\Big\} \\ &= \pi(t_r)\frac{b_0(t_r)b_0(t_s)a_1(t_r, t_s) + b_1(t_r)b_1(t_s)a_0(t_r, t_s)}{\{b_0(t_r) + b_1(t_r)\}\{b_0(t_s) + b_1(t_s)\}}. \end{aligned} \tag{B.1}$$

and substituting the parameters for the constants further gives $M_2(t_r, t_s)$ as

$$\pi(t_r)\frac{\kappa_0(t_r)\kappa_0(t_s)\mu_0^2 V_1\{\kappa_1(t_r) + \delta_1(t_r, t_s)\rho\} + \kappa_1(t_r)\kappa_1(t_s)\mu_1^2 V_0\{\kappa_0(t_r) + \delta_0(t_r, t_s)\rho\}}{2\{\kappa_0(t_r)\mu_0 + \kappa_1(t_r)\mu_1\}\{\kappa_0(t_s)\mu_0 + \kappa_1(t_s)\mu_1\}}.$$

Similarly we have

$$M_1(t_r) = \pi(t_r)\frac{\kappa_0^2(t_r)\mu_0^2 V_1\{\kappa_1(t_r) + \delta_1(t_r)\rho\} + \kappa_1^2(t_r)\mu_1^2 V_0\{\kappa_0(t_r) + \delta_0(t_r)\rho\}}{2\{\kappa_0(t_r)\mu_0 + \kappa_1(t_r)\mu_1\}^2},$$

and $M_3(t_r)$ in (13) can be shown to equal

$$M_3(t_r) = \pi(t_r)\frac{\kappa_0(t_r)\kappa_1(t_r)\mu_0\mu_1}{2\{\kappa_0(t_r)\mu_0 + \kappa_1(t_r)\mu_1\}}. \tag{B.2}$$

B.2 | Simplification of the Asymptotic Variance of $\hat{\beta}_1(t_r)$

Note that

$$\begin{aligned} \mathcal{A}^{-1}(t_r) &= \pi(t_r)^{-1}\begin{pmatrix} b_1(t_r) & b_1(t_r) \\ b_1(t_r) & b_0(t_r) + b_1(t_r) \end{pmatrix}^{-1} \\ &= \frac{\pi(t_r)^{-1}}{b_0(t_r)b_1(t_r) + b_1(t_r)^2 - b_1(t_r)^2}\begin{pmatrix} b_0(t_r) + b_1(t_r) & -b_1(t_r) \\ -b_1(t_r) & b_1(t_r) \end{pmatrix} \\ &= \pi(t_r)^{-1}\begin{pmatrix} \frac{1}{b_0(t_r)} + \frac{1}{b_1(t_r)} & -\frac{1}{b_0(t_r)} \\ -\frac{1}{b_0(t_r)} & \frac{1}{b_0(t_r)} \end{pmatrix}, \end{aligned}$$

which gives

$$\mathcal{A}^{-1}(t_r) = \pi(t_r)^{-1} \begin{pmatrix} \frac{2}{\kappa_0(t_r)\mu_0} + \frac{2}{\kappa_1(t_r)\mu_1} & -\frac{2}{\kappa_0(t_r)\mu_0} \\ -\frac{2}{\kappa_0(t_r)\mu_0} & \frac{2}{\kappa_0(t_r)\mu_0} \end{pmatrix} .$$

Then

$$\begin{aligned} \Omega(t_r) &= [\mathcal{A}^{-1}(t_r)]\,\mathcal{B}(t_r)\,[\mathcal{A}^{-1}(t_r)]' \\ &= \pi(t_r)^{-1} \begin{pmatrix} \frac{2V_0}{\kappa_0^2(t_r)\mu_0^2}[\kappa_0(t_r)+\delta_0(t_r)\rho] + \frac{2V_1}{\kappa_1^2(t_r)\mu_1^2}[\kappa_1(t_r)+\delta_1(t_r)\rho] & -\frac{2V_0}{\kappa_0^2(t_r)\mu_0^2}[\kappa_0(t_r)+\delta_0(t_r)\rho] \\ -\frac{2V_0}{\kappa_0^2(t_r)\mu_0^2}[\kappa_0(t_r)+\delta_0(t_r)\rho] & \frac{2V_0}{\kappa_0^2(t_r)\mu_0^2}[\kappa_0(t_r)+\delta_0(t_r)\rho] \end{pmatrix} . \end{aligned} \quad \text{(B.3)}$$

Thus the asymptotic variance of $\sqrt{n}(\widehat{\beta}_1(t_r) - \beta_1^\circ)$ is the (1, 1) entry of (B.3), i.e.,

$$\Omega_{\beta_1\beta_1}(t_r) = \frac{1}{\pi(t_r)} \left\{ \frac{2V_0}{\kappa_0(t_r)\mu_0^2} \left(1 + \frac{\delta_0(t_r)\rho}{\kappa_0(t_r)}\right) + \frac{2V_1}{\kappa_1(t_r)\mu_1^2} \left(1 + \frac{\delta_1(t_r)\rho}{\kappa_1(t_r)}\right) \right\} , \quad \text{(B.4)}$$

and the asymptotic variance of $\widehat{\beta}_1(t_r)$ is obtained by dividing (B.4) by n_r.

C | DERIVATIONS OF $\kappa_x(t_r)$, $\nu_x(t_r)$, AND $\kappa_x(t_r, t_s)$

Suppose K is the number of transfusions per individual over a period of time of duration τ, $K \mid u \sim \text{Poisson}(u\lambda\tau)$, and $L \mid U = u \sim \text{Exp}(u\lambda)$ and the random effect U follows the Gamma distribution with $\mathbb{E}(U) = 1$ and $\text{var}(U) = \phi$. We have

$$\begin{aligned} f(l;\lambda,\phi) &= \int_0^\infty P(l \mid u;\lambda)\mathrm{d}G(u;\phi) = \int_0^\infty u\,\lambda \exp(-u\,\lambda\,l) \frac{u^{\phi^{-1}-1} e^{-u/\phi}}{\Gamma(\phi^{-1})\phi^{\phi^{-1}}} \mathrm{d}u \\ &= \lambda \left(\frac{1}{\lambda l \phi + 1}\right)^{\phi^{-1}+1} , \end{aligned} \quad \text{(C.1)}$$

and

$$\begin{aligned} \mathrm{d}G(u \mid l;\lambda,\phi) &= \frac{P(l \mid u;\lambda)\mathrm{d}G(u;\phi)}{\int_0^\infty P(l \mid u;\lambda)\mathrm{d}G(u;\phi)} = \frac{u\,\lambda \exp(-u\,\lambda\,l) \frac{u^{\phi^{-1}-1} e^{-u/\phi}}{\Gamma(\phi^{-1})\phi^{\phi^{-1}}}}{\int_0^\infty u\,\lambda \exp(-u\,\lambda\,l) \frac{u^{\phi^{-1}-1} e^{-u/\phi}}{\Gamma(\phi^{-1})\phi^{\phi^{-1}}} \mathrm{d}u} \\ &= \frac{u^{\phi^{-1}} \exp\left(-u \,/\, \frac{\phi}{\phi\,\lambda\,l+1}\right)}{\Gamma(\phi^{-1}+1)\left(\frac{\phi}{\phi\,\lambda\,l+1}\right)^{\phi^{-1}+1}} , \end{aligned}$$

which gives

$$U \mid L = l \sim \text{Gamma}\left(\text{shape} = \phi^{-1}+1, \text{scale} = \frac{\phi}{\phi\,\lambda\,l+1}\right) .$$

To compute the $\kappa_x(t_r)$ we average over the random effect U and the recruitment time L as

$$\begin{aligned} \kappa_x(t_r) &= \mathbb{E}\{K_i(t_r) \mid X_i = x, \Delta_i(t_r) = 1\} \\ &= \mathbb{E}_L\big\{\mathbb{E}_U\big\{\mathbb{E}\big\{K_i(t_r) \mid X_i = x, \Delta_i(t_r) = 1, \\ &\qquad U_i = u, L_i = l\big\} \mid \Delta_i(t_r) = 1, L_i < \min(t_r, \tau_A)\big\} \mid \Delta_i(t_r) = 1, L_i < \min(t_r, \tau_A)\big\} . \end{aligned}$$

Note that

$$g_0(t_1,t_2) = \int_{t_1}^{t_2} f(l)\mathrm{d}l = \int_{t_1}^{t_2} \frac{\lambda}{(\lambda\phi l+1)^{\phi^{-1}+1}}\mathrm{d}l = (\lambda\phi t_1+1)^{-\phi^{-1}} - (\lambda\phi t_2+1)^{-\phi^{-1}} , \quad \text{(C.2)}$$

$$g_1(t_1,t_2) = \int_{t_1}^{t_2} lf(l)\mathrm{d}l = \int_{t_1}^{t_2} l\frac{\lambda}{(\lambda\phi l+1)^{\phi^{-1}+1}}\mathrm{d}l = \lambda\int_{t_1}^{t_2} l\,\mathrm{d}\left\{\frac{1}{-\lambda}(\lambda\phi l+1)^{-\phi^{-1}}\right\}$$
$$= \left\{-l(\lambda\phi l+1)^{-\phi^{-1}} + \frac{1}{\lambda\phi-\lambda}\,(\lambda\phi l+1)^{-\phi^{-1}+1}\right\}\Bigg|_{t_1}^{t_2}$$
$$= \frac{1}{\lambda\phi-\lambda}\left\{(\lambda\phi t_2+1)^{-\phi^{-1}+1} - (\lambda\phi t_1+1)^{-\phi^{-1}+1}\right\} - t_2(\lambda\phi t_2+1)^{-\phi^{-1}} + t_1(\lambda\phi t_1+1)^{-\phi^{-1}}, \tag{C.3}$$

and

$$g_2(t_1,t_2) = \int_{t_1}^{t_2} l^2 f(l)\mathrm{d}l = \int_{t_1}^{t_2} l^2\frac{\lambda}{(\lambda\phi l+1)^{\phi^{-1}+1}}\mathrm{d}l = \lambda\int_{t_1}^{t_2} l^2\,\mathrm{d}\left\{\frac{1}{-\lambda}(\lambda\phi l+1)^{-\phi^{-1}}\right\}$$
$$= -l^2(\lambda\phi l+1)^{-\phi^{-1}}\Big|_{t_1}^{t_2} + 2\int_{t_1}^{t_2} l(\lambda\phi l+1)^{-\phi^{-1}}\mathrm{d}l$$
$$= -l^2(\lambda\phi l+1)^{-\phi^{-1}}\Big|_{t_1}^{t_2} + 2\int_{t_1}^{t_2} l\mathrm{d}\left\{\frac{1}{-\lambda(1-\phi)}(\lambda\phi l+1)^{-\phi^{-1}+1}\right\}$$
$$= \left\{-l^2(\lambda\phi l+1)^{-\phi^{-1}} - \frac{2l}{\lambda(1-\phi)}(\lambda\phi l+1)^{-\phi^{-1}+1} - \frac{2}{\lambda^2(1-2\phi)(1-\phi)}(\lambda\phi l+1)^{-\phi^{-1}+2}\right\}\Bigg|_{t_1}^{t_2}$$
$$= -t_2^2(\lambda\phi t_2+1)^{-\phi^{-1}} + \frac{2t_2}{\lambda(\phi-1)}(\lambda\phi t_2+1)^{-\phi^{-1}+1} - \frac{2}{\lambda^2(1-2\phi)(1-\phi)}(\lambda\phi t_2+1)^{-\phi^{-1}+2}$$
$$+ t_1^2(\lambda\phi t_1+1)^{-\phi^{-1}} - \frac{2t_1}{\lambda(\phi-1)}(\lambda\phi t_1+1)^{-\phi^{-1}+1} + \frac{2}{\lambda^2(1-2\phi)(1-\phi)}(\lambda\phi t_1+1)^{-\phi^{-1}+2}. \tag{C.4}$$

Hence

$$\mathbb{E}(l \mid t_1 < l < t_2) = \frac{\int_{t_1}^{t_2} lf(l)\mathrm{d}l}{\int_{t_1}^{t_2} f(l)\mathrm{d}l} = \frac{g_1(t_1,t_2)}{g_0(t_1,t_2)}, \tag{C.5}$$
$$\mathbb{E}(l^2 \mid t_1 < l < t_2) = \frac{\int_{t_1}^{t_2} l^2 f(l)\mathrm{d}l}{\int_{t_1}^{t_2} f(l)\mathrm{d}l} = \frac{g_2(t_1,t_2)}{g_0(t_1,t_2)}. \tag{C.6}$$

Additionally,

$$\mathbb{E}\{u \mid l < t\} = \frac{\int_0^t\int_0^\infty uf(u,l)\mathrm{d}u\mathrm{d}l}{\int_0^t\int_0^\infty f(u,l)\mathrm{d}u\mathrm{d}l} = \frac{\int_0^t\int_0^\infty uf(u\mid l)f(l)\mathrm{d}u\mathrm{d}l}{\int_0^t\int_0^\infty f(u\mid l)f(l)\mathrm{d}u\mathrm{d}l}$$
$$= \frac{\int_0^t f(l)\left\{\int_0^\infty uf(u\mid l)\mathrm{d}u\right\}\mathrm{d}l}{\int_0^t f(l)\left\{\int_0^\infty f(u\mid l)\mathrm{d}u\right\}\mathrm{d}l} = \frac{\int_0^t \frac{1+\phi}{\lambda\phi l+1}f(l)\mathrm{d}l}{\int_0^t f(l)\mathrm{d}l}$$
$$= \frac{1-(\lambda\phi t+1)^{-\phi^{-1}-1}}{1-(\lambda\phi t+1)^{-\phi^{-1}}}, \tag{C.7}$$

and

$$\mathbb{E}\{u^2 \mid l < t\} = \frac{\int_0^t f(l)\left\{\int_0^\infty u^2 f(u\mid l)\mathrm{d}u\right\}\mathrm{d}l}{\int_0^t f(l)\mathrm{d}l} \tag{C.8}$$
$$= \frac{\int_0^t f(l)\left\{(\phi^{-1}+1)\left(\frac{\phi}{\lambda\phi l+1}\right)^2 + \left(\frac{1+\phi}{\lambda\phi l+1}\right)^2\right\}\mathrm{d}l}{\int_0^t f(l)\mathrm{d}l}$$
$$= \frac{(\phi+1)(2\phi+1)\int_0^t f(l)(\lambda\phi l+1)^{-2}\mathrm{d}l}{1-(\lambda\phi t+1)^{-\phi^{-1}}}$$
$$= (1+\phi)\frac{1-(\lambda\phi t+1)^{-\phi^{-1}-2}}{1-(\lambda\phi t+1)^{-\phi^{-1}}}. \tag{C.9}$$

With (C.2)-(C.8) we consider two cases for the study time t_r where 1) $0 < t_r \leq \tau_F$, 2) $\tau_F < t_r \leq t_R$.

For 1) $0 < t_r \le \tau_F$,

$$\begin{aligned}\kappa_x(t_r) &= \mathbb{E}\{K_i(t_r) \mid X_i = x, \Delta_i(t_r) = 1\}\\ &= \mathbb{E}_L\Big\{\mathbb{E}_U\Big\{\mathbb{E}\Big\{K_i(t_r) \mid X_i = x, \Delta_i(t_r) = 1,\\ &\qquad U_i = u, L_i = l\Big\} \mid \Delta_i(t_r) = 1, L_i < \min(t_r, \tau_A)\Big\} \mid \Delta_i(t_r) = 1, L_i < \min(t_r, \tau_A)\Big\}\,.\end{aligned}$$

We omit the conditions $X_i = x$ and $\Delta_i(t_r) = 1$ in the expectations for the ease of the notation so

$$\begin{aligned}\kappa_x(t_r) &= \mathbb{E}_L\left\{\mathbb{E}_U\left\{\mathbb{E}\left\{K_i(t_r) \mid U_i = u, L_i = l\right\} \mid l < \min(t_r, \tau_A)\right\} \mid l < \min(t_r, \tau_A)\right\}\\ &= \mathbb{E}_L\left\{\mathbb{E}_U\left\{u\lambda(t_r - l) \mid l < \min(t_r, \tau_A)\right\} \mid l < \min(t_r, \tau_A)\right\}\\ &= \lambda\left\{t_r - \mathbb{E}\{l \mid l < \min(t_r, \tau_A)\}\right\}\mathbb{E}\left\{u \mid l < \min(t_r, \tau_A)\right\}\\ &= \lambda\left\{t_r - \frac{g_1(0, \min(t_r, \tau_A))}{g_0(0, \min(t_r, \tau_A))}\right\}\mathbb{E}\left\{u \mid l < \min(t_r, \tau_A)\right\}\,,\end{aligned} \tag{C.10}$$

and

$$\begin{aligned}\nu_x(t_r) &= \mathrm{var}\{K_i(t_r) \mid X_i = x, \Delta_i(t_r) = 1\}\\ &= \mathbb{E}\{K_i^2(t_r) \mid X_i = x, \Delta_i(t_r) = 1\} - \kappa_x^2(t_r)\\ &= \mathbb{E}_L\left\{\mathbb{E}_U\left\{\mathbb{E}\left\{K_i^2(t_r) \mid U_i = u, L_i = l\right\} \mid l < \min(t_r, \tau_A)\right\} \mid l < \min(t_r, \tau_A)\right\} - \kappa_x^2(t_r)\\ &= \mathbb{E}_L\Big\{\mathbb{E}_U\Big\{\mathrm{var}\Big\{K_i(t_r) \mid U_i = u, L_i = l\Big\}\\ &\qquad + \mathbb{E}^2\Big\{K_i(t_r) \mid U_i = u, L_i = l\Big\} \mid l < \min(t_r, \tau_A)\Big\} \mid l < \min(t_r, \tau_A)\Big\} - \kappa_x^2(t_r)\\ &= \mathbb{E}_L\left\{\mathbb{E}_U\left\{u\lambda(t_r - l) + u^2\lambda^2(t_r - l)^2 \mid l < \min(t_r, \tau_A)\right\} \mid l < \min(t_r, \tau_A)\right\} - \kappa_x^2(t_r)\\ &= \kappa_x(t_r) + \lambda^2\{t_r^2 + \mathbb{E}\{l^2 \mid l < \min(t_r, \tau_A)\} - 2t_r\mathbb{E}\{l \mid l < \min(t_r, \tau_A)\}\}\mathbb{E}\{u^2 \mid l < \min(t_r, \tau_A)\} - \kappa_x^2(t_r)\\ &= \kappa_x(t_r) + \lambda^2\left\{t_r^2 + \frac{g_2(0, \min(t_r, \tau_A))}{g_0(0, \min(t_r, \tau_A))} - 2t_r\frac{g_1(0, \min(t_r, \tau_A))}{g_0(0, \min(t_r, \tau_A))}\right\}\mathbb{E}\left\{u^2 \mid l < \min(t_r, \tau_A)\right\} - \kappa_x^2(t_r)\,.\end{aligned} \tag{C.11}$$

For 2) $\tau_F < t_r \le t_R$ we have

$$\begin{aligned}\kappa_x(t_r) &= \mathbb{E}\{K_i(t_r) \mid X_i = x, \Delta_i(t_r) = 1\}\\ &= \mathbb{E}_L\left\{\mathbb{E}_U\left\{\mathbb{E}\left\{K_i(t_r) \mid U_i = u, L_i = l\right\} \mid l < \min(t_r, \tau_A)\right\} \mid l < \min(t_r, \tau_A)\right\}\\ &= \mathbb{E}_L\left\{\mathbb{E}_U\left\{u\lambda\min(t_r - l, \tau_F) \mid l < \min(t_r, \tau_A)\right\} \mid l < \min(t_r, \tau_A)\right\}\\ &= \lambda\mathbb{E}\left\{u \mid l < \min(t_r, \tau_A)\right\}\mathbb{E}_L\left\{\min(t_r - l, \tau_F) \mid l < \min(t_r, \tau_A)\right\}\\ &= \lambda\mathbb{E}\left\{u \mid l < \min(t_r, \tau_A)\right\}\left\{\frac{\int_{t_r-\tau_F}^{\min(t_r,\tau_A)}(t_r - l)f(l)\mathrm{d}l + \int_0^{t_r-\tau_F}\tau_F f(l)\mathrm{d}l}{g_0(0, \min(t_r, \tau_A))}\right\}\\ &= \lambda\mathbb{E}\left\{u \mid l < \min(t_r, \tau_A)\right\}\left\{t_r\frac{g_0(t_r - \tau_F, \min(t_r, \tau_A))}{g_0(0, \min(t_r, \tau_A))} - \frac{g_1(t_r - \tau_F, \min(t_r, \tau_A))}{g_0(0, \min(t_r, \tau_A))} + \tau_F\frac{g_0(0, t_r - \tau_F)}{g_0(0, \min(t_r, \tau_A))}\right\}\,,\end{aligned} \tag{C.12}$$

and

$$
\begin{aligned}
\nu_x(t_r) &= \mathrm{var}\{K_i(t_r) \mid X_i = x, \Delta_i(t_r) = 1\} \\
&= \mathbb{E}\{K_i^2(t_r) \mid X_i = x, \Delta_i(t_r) = 1\} - \kappa_x^2(t_r) \\
&= \mathbb{E}_L\left\{\mathbb{E}_U\left\{\mathbb{E}\left\{K_i^2(t_r) \mid U_i = u, L_i = l\right\} \mid l < \min(t_r, \tau_A)\right\} \mid l < \min(t_r, \tau_A)\right\} - \kappa_x^2(t_r) \\
&= \mathbb{E}_L\Big\{\mathbb{E}_U\Big\{\mathrm{var}\Big\{K_i(t_r) \mid U_i = u, L_i = l\Big\} \\
&\qquad + \mathbb{E}^2\Big\{K_i(t_r) \mid U_i = u, L_i = l\Big\} \mid l < \min(t_r, \tau_A)\Big\} \mid l < \min(t_r, \tau_A)\Big\} - \kappa_x^2(t_r) \\
&= \mathbb{E}_L\left\{\mathbb{E}_U\left\{u\lambda \min(t_r - l, \tau_F) + u^2\lambda^2(\min(t_r - l, \tau_F))^2 \mid l < \min(t_r, \tau_A)\right\} \mid l < \min(t_r, \tau_A)\right\} - \kappa_x^2(t_r) \\
&= \kappa_x(t_r) + \lambda^2\mathbb{E}\left\{u^2 \mid l < \min(t_r, \tau_A)\right\} \mathbb{E}_L\left\{(\min(t_r - l, \tau_F))^2 \mid l < \min(t_r, \tau_A)\right\} - \kappa_x^2(t_r) \\
&= \kappa_x(t_r) + \lambda^2\mathbb{E}\left\{u^2 \mid l < \min(t_r, \tau_A)\right\} \left\{\frac{\int_{t_r-\tau_F}^{\min(t_r,\tau_A)}(t_r - l)^2 f(l)\,\mathrm{d}l + \int_0^{t_r-\tau_F} \tau_F^2 f(l)\mathrm{d}l}{g_0(0, \min(t_r, \tau_A))}\right\} - \kappa_x^2(t_r) \\
&= \kappa_x(t_r) + \lambda^2\mathbb{E}\left\{u^2 \mid l < \min(t_r, \tau_A)\right\} \left\{\frac{1}{g_0(0, \min(t_r, \tau_A))}\left\{\int_{t_r-\tau_F}^{\min(t_r,\tau_A)} t_r^2 f(l)\mathrm{d}l - \int_{t_r-\tau_F}^{\min(t_r,\tau_A)} 2t_r\, l f(l)\mathrm{d}l\right.\right. \\
&\qquad \left.\left. + \int_{t_r-\tau_F}^{\min(t_r,\tau_A)} l^2 f(l)\,\mathrm{d}l + \int_0^{t_r-\tau_F} \tau_F^2 f(l)\,\mathrm{d}l\right\}\right\} - \kappa_x^2(t_r) \\
&= \kappa_x(t_r) + \lambda^2\mathbb{E}\left\{u^2 \mid l < \min(t_r, \tau_A)\right\} \left\{\frac{1}{g_0(0, \min(t_r, \tau_A))}\left\{t_r^2 g_0(t_r - \tau_F, \min(t_r, \tau_A)) - 2t_r g_1(t_r - \tau_F, \min(t_r, \tau_A))\right.\right. \\
&\qquad \left.\left. + g_2(t_r - \tau_F, \min(t_r, \tau_A)) + \tau_F^2 g_0(0, t_r - \tau_F)\right\}\right\} - \kappa_x^2(t_r) \\
&= \kappa_x(t_r) + \lambda^2\mathbb{E}\left\{u^2 \mid l < \min(t_r, \tau_A)\right\} \left\{t_r^2 \frac{g_0(t_r - \tau_F, \min(t_r, \tau_A))}{g_0(0, \min(t_r, \tau_A))} - 2t_r \frac{g_1(t_r - \tau_F, \min(t_r, \tau_A))}{g_0(0, \min(t_r, \tau_A))}\right. \\
&\qquad \left. + \frac{g_2(t_r - \tau_F, \min(t_r, \tau_A))}{g_0(0, \min(t_r, \tau_A))} + \tau_F^2 \frac{g_0(0, t_r - \tau_F)}{g_0(0, \min(t_r, \tau_A))}\right\} - \kappa_x^2(t_r)\,. \qquad \text{(C.13)}
\end{aligned}
$$

To compute $\kappa_x(t_r, t_s)$ we consider three cases 1) $0 < t_r < t_s \leq \tau_F$, 2) $0 < t_r < \tau_F < t_s \leq t_R$, 3) $\tau_F \leq t_r < t_s \leq t_R$. For 1) $0 < t_r < t_s \leq \tau_F$,

$$
\begin{aligned}
\kappa_x(t_r, t_s) &= \mathbb{E}\{K_i(t_r, t_s) \mid X_i = x, \Delta_i(t_r) = 1\} \\
&= \mathbb{E}_L\left\{\mathbb{E}_U\left\{\mathbb{E}\left\{K_i(t_r, t_s) \mid U_i = u, L_i = l\right\} \mid l < \min(t_r, \tau_A)\right\} \mid l < \min(t_r, \tau_A)\right\} \\
&= \mathbb{E}_L\left\{\mathbb{E}_U\left\{u\lambda(t_s - t_r) \mid l < \min(t_r, \tau_A)\right\} \mid l < \min(t_r, \tau_A)\right\} \\
&= \lambda(t_s - t_r)\mathbb{E}\left\{u \mid l < \min(t_r, \tau_A)\right\}\,. \qquad \text{(C.14)}
\end{aligned}
$$

For 2) $0 < t_r < \tau_F < t_s \leq t_R$,

$$
\begin{aligned}
\kappa_x(t_r, t_s) &= \mathbb{E}\{K_i(t_r, t_s) \mid X_i = x, \Delta_i(t_r) = 1\} \\
&= \mathbb{E}_L\left\{\mathbb{E}_U\left\{\mathbb{E}\left\{K_i(t_r, t_s) \mid U_i = u, L_i = l\right\} \mid l < \min(t_r, \tau_A)\right\} \mid l < \min(t_r, \tau_A)\right\} \\
&= \mathbb{E}_L\left\{\mathbb{E}_U\left\{u\lambda \min(t_s - t_r, l + \tau_F - t_r) \mid l < \min(t_r, \tau_A)\right\} \mid l < \min(t_r, \tau_A)\right\} \\
&= \lambda\mathbb{E}\left\{u \mid l < \min(t_r, \tau_A)\right\} \mathbb{E}_L\left\{\min(t_s - t_r, l + \tau_F - t_r) \mid l < \min(t_r, \tau_A)\right\} \\
&= \lambda\mathbb{E}\left\{u \mid l < \min(t_r, \tau_A)\right\} \frac{\int_{t_s-\tau_F}^{\min(t_r,\tau_A)}(t_s - t_r) f(l)\,\mathrm{d}l + \int_0^{t_s-\tau_F}(l + \tau_F - t_r) f(l)\,\mathrm{d}l}{g_0(0, \min(t_r, \tau_A))} \\
&= \lambda\mathbb{E}\left\{u \mid l < \min(t_r, \tau_A)\right\} \frac{(t_s - t_r) g_0(t_s - \tau_F, \min(t_r, \tau_A)) + g_1(0, t_s - \tau_F) + (\tau_F - t_r) g_0(0, t_s - \tau_F)}{g_0(0, \min(t_r, \tau_A))} \\
&= \lambda\mathbb{E}\left\{u \mid l < \min(t_r, \tau_A)\right\} \left\{(t_s - t_r)\frac{g_0(t_s - \tau_F, \min(t_r, \tau_A))}{g_0(0, \min(t_r, \tau_A))} + \frac{g_1(0, t_s - \tau_F)}{g_0(0, \min(t_r, \tau_A))} + (\tau_F - t_r)\frac{g_0(0, t_s - \tau_F)}{g_0(0, \min(t_r, \tau_A))}\right\}. \qquad \text{(C.15)}
\end{aligned}
$$

For 3) $\tau_F \leq t_r < t_s \leq t_R$,

$$\begin{aligned}
\kappa_x(t_r, t_s) &= \mathbb{E}\{K_i(t_r, t_s) \mid X_i = x, \Delta_i(t_r) = 1\} \\
&= \mathbb{E}_L \{\mathbb{E}_U \{\mathbb{E}\{K_i(t_r, t_s) \mid U_i = u, L_i = l\} \mid l < \min(t_r, \tau_A)\} \mid l < \min(t_r, \tau_A)\} \\
&= \mathbb{E}_L \{\mathbb{E}_U \{u\lambda \max(\min(t_s - t_r, l + \tau_F - t_r), 0) \mid l < \min(t_r, \tau_A)\} \mid l < \min(t_r, \tau_A)\} \\
&= \lambda\mathbb{E}\{u \mid l < \min(t_r, \tau_A)\}\, \mathbb{E}_L \{\max(\min(t_s - t_r, l + \tau_F - t_r), 0) \mid l < \min(t_r, \tau_A)\} \\
&= \lambda\mathbb{E}\{u \mid l < \min(t_r, \tau_A)\} \left\{ \frac{\int_{t_s-\tau_F}^{\min(t_r,\tau_A)} (t_s - t_r) f(l)\, \mathrm{d}l + \int_0^{t_s-\tau_F} (l + \tau_F - t_r) f(l)\, \mathrm{d}l}{g_0(0, \min(t_r, \tau_A))} \right\} \\
&= \lambda\mathbb{E}\{u \mid l < \min(t_r, \tau_A)\} \frac{(t_s - t_r) g_0(t_s - \tau_F, \min(t_r, \tau_A)) + g_1(0, t_s - \tau_F) + (\tau_F - t_r) g_0(0, t_s - \tau_F)}{g_0(0, \min(t_r, \tau_A))} \\
&= \lambda\mathbb{E}\{u \mid l < \min(t_r, \tau_A)\} \left\{ (t_s - t_r) \frac{g_0(t_s - \tau_F, \min(t_r, \tau_A))}{g_0(0, \min(t_r, \tau_A))} + \frac{g_1(0, t_s - \tau_F)}{g_0(0, \min(t_r, \tau_A))} + (\tau_F - t_r) \frac{g_0(0, t_s - \tau_F)}{g_0(0, \min(t_r, \tau_A))} \right\}, \qquad \text{(C.16)}
\end{aligned}$$

which is the same as the case when $0 < t_r < \tau_F < t_s \leq t_R$ in 2). Additionally from (C.1) we have

$$\begin{aligned}
\pi(t_r) &= P(\Delta_i(t_r) = 1) = P(l_i \leq t_r \mid l_i \leq \tau_A) \\
&= \frac{\int_0^{t_r} \lambda(\lambda l\phi + 1)^{-\phi^{-1}-1} \mathrm{d}l}{\int_0^{\tau_A} \lambda(\lambda l\phi + 1)^{-\phi^{-1}-1} \mathrm{d}l} = \frac{\lambda \left(\frac{1}{-\lambda} (\lambda l\phi + 1)^{-\phi^{-1}} \right) \Big|_0^{t_r}}{\lambda \left(\frac{1}{-\lambda} (\lambda l\phi + 1)^{-\phi^{-1}} \right) \Big|_0^{\tau_A}} = \frac{1 - (\lambda\phi t_r + 1)^{-\phi^{-1}}}{1 - (\lambda\phi\tau_A + 1)^{-\phi^{-1}}}.
\end{aligned}$$

D | SUPPLEMENTARY TABLE

Table D.1 presents the one-sided boundaries for a non-inferiority trial design of a plasma donation study as discussed in Section 5.3. The parameter settings are as follows, $\lambda = 21$, $\phi = 0.8$, $\mu_0 = 0.035\%$, $\exp(\beta_{1A}) = 1.5$, $\exp(\beta_{1M}) = 2.5$, $\rho = 0.005$, $\tau_A = 0.5$, $\tau_F = 0.5$, $\alpha = 5\%$, and target power=80%. Boundaries based on O'Brien & Fleming, Pocock, and uniform-type alpha spending functions are listed. Two interim analyses with one final analysis are planned; 1st interim analysis is planned at study time t_1, 2nd interim analysis is planned at study time t_2, and the final analysis is planned at the end of the study. The information fraction is determined as the fraction of inverse variance of $\hat{\beta}_1$.

TABLE D.1 One-sided boundaries from different alpha spending functions for a non-inferiority trial with 1st interim analysis planned at study time t_1, 2nd interim analysis planned at study time t_2, and final analysis planned at the end of study; information fraction is determined as the fraction of inverse variance of $\hat{\beta}_1$; $\lambda = 21$, $\phi = 0.8$, $\mu_0 = 0.035\%$, $\exp(\beta_{1A}) = 1.5$, $\exp(\beta_{1M}) = 2.5$, $\rho = 0.005$, $\tau_A = 0.5$, $\tau_F = 0.5$, $\alpha = 5\%$, and target power=80%.

(a) O'Brien & Fleming Type ($\alpha_1^*(t)$)

1st interim analysis at t_1	2nd interim analysis at t_2: 0.1	0.2	0.3	0.4	0.5	0.6	0.7	0.8	0.9	1.0
**0.1**	(5.08, –, 1.64)	(5.08, 3.25, 1.65)	(5.08, 2.51, 1.66)	(5.08, 2.09, 1.70)	(5.08, 1.81, 1.75)	(5.08, 1.70, 1.78)	(5.08, 1.67, 1.84)	(5.08, 1.65, 1.96)	(5.08, 1.65, 2.15)	(5.08, 1.64, –)
**0.2**	–	(3.25, –, 1.65)	(3.25, 2.52, 1.66)	(3.25, 2.09, 1.70)	(3.25, 1.81, 1.75)	(3.25, 1.70, 1.78)	(3.25, 1.67, 1.84)	(3.25, 1.65, 1.96)	(3.25, 1.65, 2.15)	(3.25, 1.65, –)
**0.3**	–	–	(2.51, –, 1.66)	(2.51, 2.13, 1.70)	(2.51, 1.83, 1.76)	(2.51, 1.72, 1.79)	(2.51, 1.69, 1.85)	(2.51, 1.67, 1.97)	(2.51, 1.66, 2.16)	(2.51, 1.66, –)
**0.4**	–	–	–	(2.09, –, 1.70)	(2.09, 1.88, 1.77)	(2.09, 1.76, 1.81)	(2.09, 1.72, 1.87)	(2.09, 1.71, 1.99)	(2.09, 1.70, 2.18)	(2.09, 1.70, –)
**0.5**	–	–	–	–	(1.81, –, 1.75)	(1.81, 1.81, 1.82)	(1.81, 1.77, 1.88)	(1.81, 1.76, 1.99)	(1.81, 1.75, 2.17)	(1.81, 1.75, –)
**0.6**	–	–	–	–	–	(1.70, –, 1.78)	(1.70, 1.81, 1.85)	(1.70, 1.79, 1.94)	(1.70, 1.78, 2.09)	(1.70, 1.78, –)
**0.7**	–	–	–	–	–	–	(1.67, –, 1.84)	(1.67, 1.86, 1.92)	(1.67, 1.84, 2.06)	(1.67, 1.84, –)
**0.8**	–	–	–	–	–	–	–	(1.65, –, 1.96)	(1.65, 1.97, 2.08)	(1.65, 1.96, –)
**0.9**	–	–	–	–	–	–	–	–	(1.65, –, 2.15)	(1.65, 2.15, –)
**1.0**	–	–	–	–	–	–	–	–	–	(1.64, –, –)

(b) Pocock Type ($\alpha_2^*(t)$)

1st interim analysis at t_1	2nd interim analysis at t_2: 0.1	0.2	0.3	0.4	0.5	0.6	0.7	0.8	0.9	1.0
**0.1**	(2.29, –, 1.73)	(2.29, 2.17, 1.83)	(2.29, 1.98, 1.91)	(2.29, 1.86, 1.96)	(2.29, 1.78, 1.99)	(2.29, 1.75, 1.97)	(2.29, 1.74, 2.02)	(2.29, 1.73, 2.14)	(2.29, 1.73, 2.33)	(2.29, 1.73, –)
**0.2**	–	(2.01, –, 1.82)	(2.01, 2.09, 1.91)	(2.01, 1.96, 1.98)	(2.01, 1.87, 2.03)	(2.01, 1.83, 2.03)	(2.01, 1.82, 2.08)	(2.01, 1.82, 2.20)	(2.01, 1.82, 2.39)	(2.01, 1.82, –)
**0.3**	–	–	(1.86, –, 1.88)	(1.86, 2.03, 1.98)	(1.86, 1.94, 2.05)	(1.86, 1.90, 2.07)	(1.86, 1.89, 2.12)	(1.86, 1.89, 2.24)	(1.86, 1.88, 2.42)	(1.86, 1.88, –)
**0.4**	–	–	–	(1.76, –, 1.93)	(1.76, 1.97, 2.03)	(1.76, 1.95, 2.08)	(1.76, 1.94, 2.14)	(1.76, 1.93, 2.25)	(1.76, 1.93, 2.43)	(1.76, 1.93, –)
**0.5**	–	–	–	–	(1.69, –, 1.93)	(1.69, 1.93, 2.02)	(1.69, 1.94, 2.08)	(1.69, 1.93, 2.18)	(1.69, 1.93, 2.35)	(1.69, 1.93, –)
**0.6**	–	–	–	–	–	(1.66, –, 1.91)	(1.66, 1.90, 1.98)	(1.66, 1.91, 2.06)	(1.66, 1.91, 2.20)	(1.66, 1.91, –)
**0.7**	–	–	–	–	–	–	(1.65, –, 1.95)	(1.65, 1.96, 2.03)	(1.65, 1.96, 2.15)	(1.65, 1.95, –)
**0.8**	–	–	–	–	–	–	–	(1.65, –, 2.07)	(1.65, 2.08, 2.18)	(1.65, 2.07, –)
**0.9**	–	–	–	–	–	–	–	–	(1.65, –, 2.26)	(1.65, 2.26, –)
**1.0**	–	–	–	–	–	–	–	–	–	(1.64, –, –)

(c) Uniform Type ($\alpha_3^*(t)$)

1st interim analysis at t_1	2nd interim analysis at t_2: 0.1	0.2	0.3	0.4	0.5	0.6	0.7	0.8	0.9	1.0
**0.1**	(2.45, –, 1.70)	(2.45, 2.27, 1.76)	(2.45, 2.04, 1.82)	(2.45, 1.89, 1.87)	(2.45, 1.77, 1.90)	(2.45, 1.72, 1.90)	(2.45, 1.71, 1.96)	(2.45, 1.70, 2.07)	(2.45, 1.70, 2.26)	(2.45, 1.70, –)
**0.2**	–	(2.14, –, 1.75)	(2.14, 2.14, 1.82)	(2.14, 1.96, 1.88)	(2.14, 1.83, 1.93)	(2.14, 1.78, 1.94)	(2.14, 1.77, 2.00)	(2.14, 1.76, 2.11)	(2.14, 1.76, 2.30)	(2.14, 1.75, –)
**0.3**	–	–	(1.95, –, 1.81)	(1.95, 2.03, 1.88)	(1.95, 1.89, 1.95)	(1.95, 1.84, 1.97)	(1.95, 1.82, 2.03)	(1.95, 1.81, 2.14)	(1.95, 1.81, 2.33)	(1.95, 1.81, –)
**0.4**	–	–	–	(1.82, –, 1.85)	(1.82, 1.93, 1.94)	(1.82, 1.88, 1.98)	(1.82, 1.86, 2.04)	(1.82, 1.85, 2.15)	(1.82, 1.85, 2.34)	(1.82, 1.85, –)
**0.5**	–	–	–	–	(1.71, –, 1.87)	(1.71, 1.89, 1.95)	(1.71, 1.88, 2.01)	(1.71, 1.87, 2.11)	(1.71, 1.87, 2.28)	(1.71, 1.87, –)
**0.6**	–	–	–	–	–	(1.67, –, 1.87)	(1.67, 1.86, 1.93)	(1.67, 1.87, 2.02)	(1.67, 1.87, 2.16)	(1.67, 1.87, –)
**0.7**	–	–	–	–	–	–	(1.65, –, 1.92)	(1.65, 1.93, 2.00)	(1.65, 1.92, 2.12)	(1.65, 1.92, –)
**0.8**	–	–	–	–	–	–	–	(1.65, –, 2.03)	(1.65, 2.04, 2.15)	(1.65, 2.03, –)
**0.9**	–	–	–	–	–	–	–	–	(1.65, –, 2.22)	(1.65, 2.22, –)
**1.0**	–	–	–	–	–	–	–	–	–	(1.64, –, –)
